# Blockchain-enhanced electoral integrity: a robust model for secure digital voting systems in Oman

**DOI:** 10.12688/f1000research.160087.1

**Published:** 2025-02-19

**Authors:** Abdul Shaikh, Naresh Adhikari, Amril Nazir, Abdul Salam Shah, Saranjam Baig, Hafedh Al Shihi

**Affiliations:** 1 Information Systems, Sultan Qaboos University, Muscat, Muscat Governorate, 123, Oman; 2 Department of Computer Science, Slippery Rock University, Slippery Rock, Pennsylvania, 16057, USA; 3 Information Systems and Technology Management, Zayed University, Abu Dhabi, Abu Dhabi, 144534, United Arab Emirates; 4 School of Computer Science, Faculty of Innovation and Technology, Taylor's University, Subang Jaya, Selangor, Malaysia; 5 College of Economic and Political Science, Sultan Qaboos University, Muscat, Muscat Governorate, 123, Oman

**Keywords:** Blockchain Technology, Electoral Integrity, Digital Voting Systems, Voter Security, Transparent Elections, Secure Voter Authentication, Decentralized voting, Oman

## Abstract

**Background:**

Ensuring the security and trustworthiness of a digitized and automated electoral process remains a significant challenge in democratic systems. As digital voting systems are increasingly being investigated around the world, ensuring the integrity of the process using robust security measures is of great importance. This paper presents a simplified model to enhance electoral integrity by leveraging Blockchain technology in the context of Oman’s digital voting system. The model uses Blockchain technology to create a secure and trustworthy voting environment, addressing key vulnerabilities in digital electoral systems.

**Methods:**

The research utilized a quantitative approach, employing an experimental design methodology using open-source software to simulate voting systems. Synthetic population data is utilized for operating these systems, while advanced biometric authentication technologies are used to verify voter identities. Blockchain technology is leveraged to ensure secure vote recording, with smart contracts used to authenticate voters and securely record votes. Additionally, synchronous transactions are executed for both voter registration and voting processes, enhancing the overall security and efficiency of the system.

**Results:**

The experimental results shows that Blockchain enhances electoral integrity and security in Oman voting system, improves transparency and reliability in elections. The performance evaluation of the model focuses on efficiency, reliability, and scalability metrics. Asynchronous transactions are utilized to improve processing time for voter registration and voting. Election administrators can manage, monitor, and certify election results, while Ethereum nodes ensure decentralized verification and transparency in the voting process.

**Conclusion:**

This research offers insights for policymakers to consider Blockchain for electoral reforms, addressing issues like data integrity, fraud prevention, and transparency to boost voter trust. A strong regulatory framework and public awareness are crucial for successful implementation. Pilot projects are needed to assess Blockchain’s practical impact. Oman could lead global innovation in electoral technology, though infrastructure and public resistance challenges must be managed.

## 1. Introduction

Governments around the world use voting to implement democracy, as elections are the backbone of democracy that showcases the views of citizens in terms of votes. ^1^ A vote is a formal expression of a choice made between two or more candidates or courses of action. This process typically involves using a ballot, a show of hands, or other methods to indicate one’s preference. ^2^ Voting is the foundation of any effective democracy; therefore, it must be accessible and secure for all citizens. Ensuring that every qualified individual can participate in the voting process is vital to preserving the integrity and legitimacy of democratic systems. ^3^ It ensures that all perspectives are taken into account, strengthens equity and justice, and improves public confidence in the electoral process. ^4^ Traditional voting was often conducted by displaying thumbs-ups (approval) or thumbs-downs (disapproval) from the voters. The candidate who received the highest number of thumbs-up was declared the winner. This straightforward method of voting allowed for open and immediate visible feedback, making it easy to determine the outcome. ^5^ This simple approach was effective in small gatherings or assemblies where decisions needed to be made quickly and transparently. ^6^ The electoral system must have ultimate safety to ensure access to legitimate users to avoid any inside and outside interference that can prevent votes from being cast or prevent the voter’s vote from being altered. ^7^ Bracamonte et al. assert that a successful voting system meets the criteria of the electoral system that optimize the advantages of the system for the citizens and the administration. ^8^

There are several challenges associated with traditional and digital voting methods. Firstly, security concerns are significant, as these methods are susceptible to manipulation, fraud, and cyber attacks, raising doubts about the integrity of election outcomes. ^9^ Secondly, traditional voting lacks transparency, leading to questions about the legitimacy of results due to opaque processes. In addition, accessibility remains an issue for certain demographics, such as individuals with disabilities or those in remote locations, who may face barriers to participating in the voting process. ^10^ In addition, the high costs associated with physical infrastructure, printed ballots, and manpower contribute to the cost of elections. In addition, manual or electronic vote counting processes can be time consuming, resulting in delays in announcing results. Finally, factors such as inconvenient polling locations, long waiting times, or limited voting hours can discourage voter turnout, undermining the democratic process. ^11– 13^ Addressing these challenges is crucial to improving the efficiency, security, and inclusivity of elections, with Blockchain technology offering potential solutions to many of these issues.

Blockchain technology comprises the exchange and storage of verified transactions over a distributed network of computing nodes. The technology leverages techniques that ensure the integrity and security of data by maintaining a distributed ledger, also known as a Blockchain, which is a series of interlinked data records. Each transaction or record, once added to the blockchian, is linked to the previous entry using the previous block’s one-way hash functions, forming a chronological chain of blocks. ^14^ The Blockchains are maintained across a network of computers, also known as qualifying nodes.

The decentralized verification of transactions before they are added to the temper-proof Blockchain eliminates the need for a central authority, as all participants in the network have access to the same information, ensuring transparency and reducing the risk of fraud such as double spending. ^15^ The technology involves complex inter-process communication that verifies and validates each transaction, making it nearly impossible to alter or tamper once the transaction is added to the Blockchain. ^16^ For instance, Bitcoin, created by Satoshi Nakamoto in 2008, is a popular application of Blockchain technology that facilitates payments of transactions between two parties without any intermediary, such as credit card companies and banks. ^16^

The proposed electronic voting model leverages blockchain technology to enhance security, transparency, and trust in the entire electoral process. This research considers the case of Oman to present traditional and electronic systems in the election process, which is reflected in the electronic voting system used in recent Omani elections held on 27 ^th^ October 2019. The contributions of this research are as follows:

• Identify and discuss security and trust issues with traditional electoral systems.
• Propose and prototype a simplified blockchain-based electoral system.
• Implement and test the smart-contracts required for blockchain-based electoral system.
• Evaluate proposed digital blockchain-based electoral system in terms of performance matrices such as time required for registration and voting.

The rest of the paper is organized as Section 2 discusses the preliminaries, Section 3 presents related works published on the Blockchain and electronic voting, and Section 4 contains system models and methods used in the proposed model. Section 5 presents the experimental setup of the study while Section 6 presents a detailed discussion of the results followed by Section 7 conclusion.

## 2. Preliminaries

### 2.1 The Omani Election System: An overview

There are two main councils in Oman, Majlis Al-Shura (Lower House) and Majlis Al Dawla (Upper House). These two councils are under the name of “Majlis Oman”. Majlis Al Dawla members were appointed previously by His Majesty Sultan Qaboos and currently by His Majesty Sultan Haitham, from previous government officials and prominent citizens. On the other hand, Majlis Al-Shura members are chosen by the citizens via voting, but this is not mandatory. Majlis Al-Shura was founded in 1991, as a substitute for the traditional consultative council of the state. In Oman, there are 61 Wilayats (administrative division). Each member of the Majlis represents one Wilayat, and if the population of the Wilayat exceeds 30 *,* 000 people, two members represent that Wilayat. The membership term in Majlis Al-Shura is four years. ^17^

The Ministry of Interior (MoI) is responsible for the elections in Oman. The voter has to first register at the MoI to have the right to cast a vote. There are some conditions for voters to be eligible to vote ^18^:

• Must have Omani nationality.
• Must be 21 years old or above.
• Must cast the vote in the voter’s Wilayat or the address written on the identity card (ID) of the voter.

On the day of the election, each voter casts his/her vote at the election station located in his/her Wilayat. In the election center, the verification is done through the ID of the person manually by the administration committee stationed at the election station. After the verification has been done successfully, the voter is given the voting paper in which the voter chooses and marks the candidate of his/her choice and drops the paper in the selection box. Each voter has the right to cast a vote only once and for a single candidate. At the end of the election, the election audit committee at each station reviews all voting ballots manually and calculates the election results. This lengthy process comes with several drawbacks such as:

• The voter has to visit the election station physically to be able to cast a vote (which depends on the location of Wilayat).
• The results have to be calculated manually, which is time-consuming and is prone to many problems such as manipulation, missing to count, among others.
• There is a high possibility of fraud which can be perceived during the calculation of the result.

**Figure 1.**
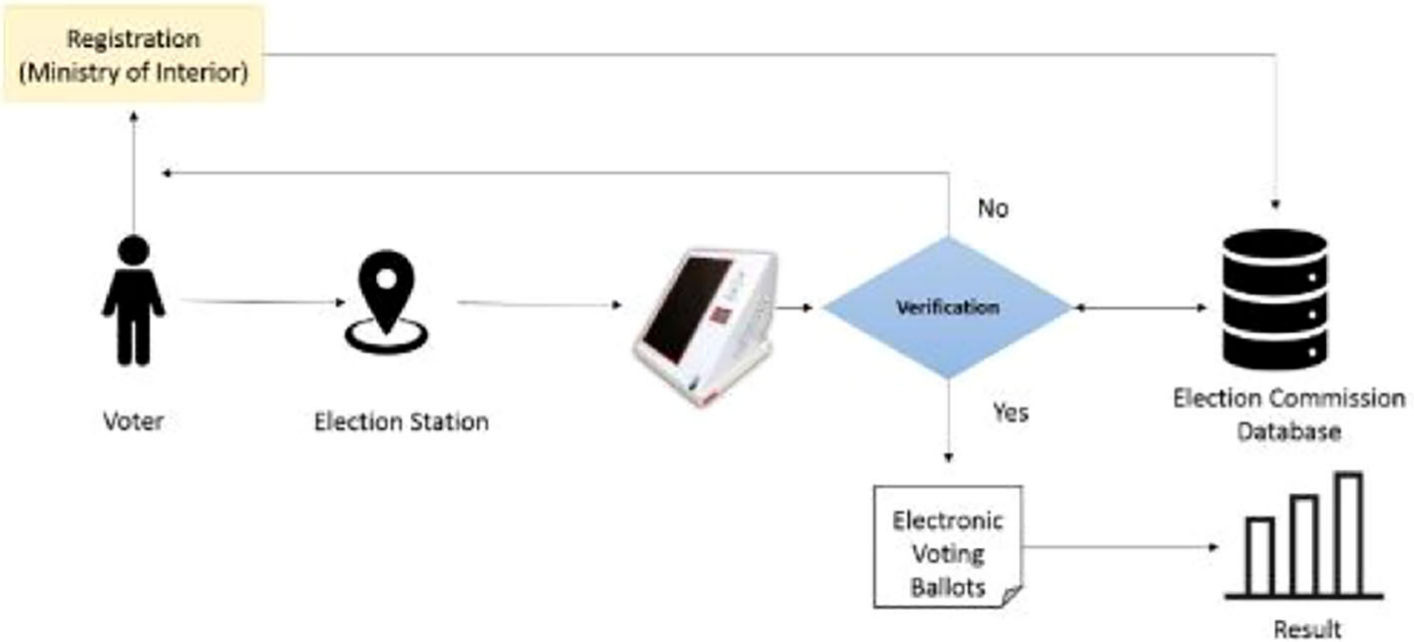
Use case model of the system.

However, traditional voting, which depended on paper and pen, no longer exists in Oman. Oman has implemented the electronic voting system to improve and improve the electoral process by keeping it more traceable and verifiable. The 9 ^th^ general election in Oman, held on 27 ^th^ 2019, was conducted using an electronic voting system. The electronic election is executed through an electronic device called’Sawtak’ that is distributed at each election station. It allows the verification process automatically once the user enters his/her ID after which the voter has to do the fingerprint authentication process. If the user is verified successfully, then the electronic ballot list of the candidates is displayed for 120 seconds for selection. This list is not available to unauthorized users such as unregistered users from the MoI (Ministry of Interior) and users who try to access the list from different election stations. Once the list appears and the voter picks a candidate, an electronic ticket is displayed on the device that includes the username and the chosen candidate to verify the completion of the electoral process. The researcher illustrates the process of the electronic election in Oman in Figure 1.

### 2.2 Blockchain

**Figure 2.**
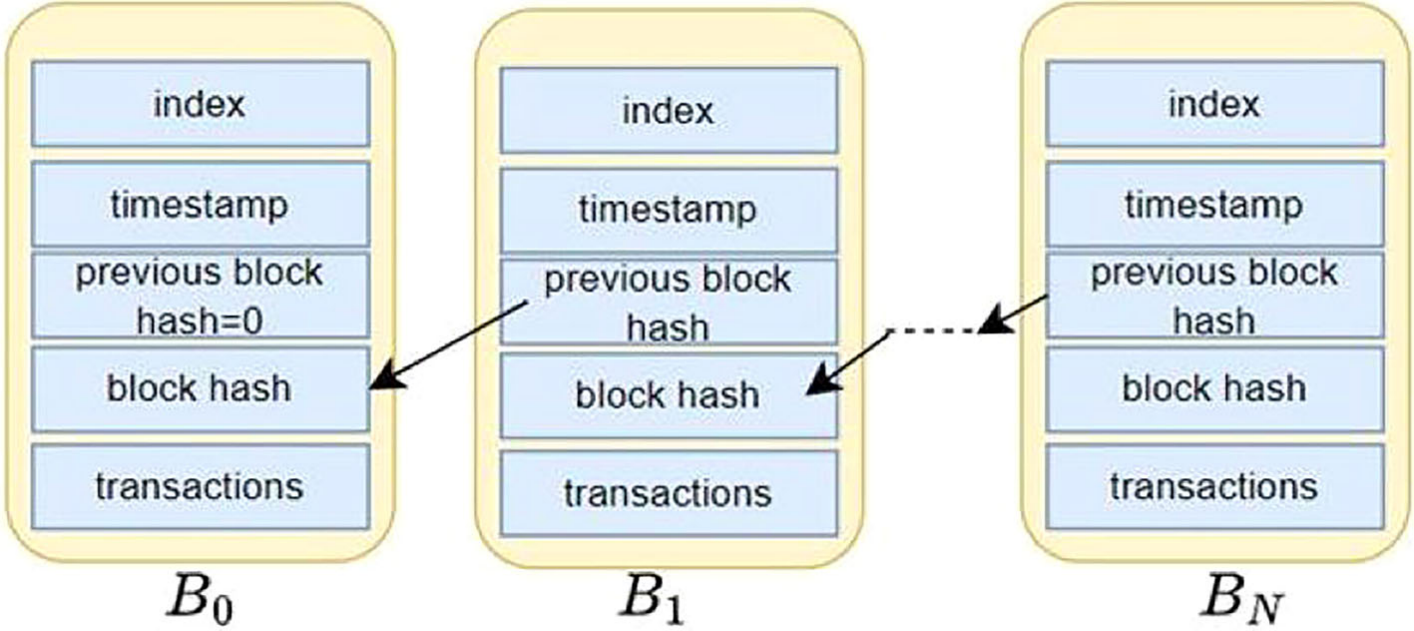
Structure of a simple Blockchain ledger. ^19^ This figure has been reproduced with permission from Adhikari et al. (2023). ^19^

Blockchain may be described as a chain of data blocks that represent an ordered storage of the blocks. ^19^ Each data block within the chain is connected to the preceding block with the help of containing a cryptographic reference to the previous block. The cryptographic reference, additionally referred to as block hash, is usually the secure hash value of the contents within the preceding block. If *M* are data contained in a block, then *δ* = *H*( *M*) is the block hash, where *H*( *.*) is a secure, one-way function such as SHA-2, SHA-3, among others. A block can contain different information such as block index, timestamp, previous block’s block hash, number of transactions in the block, and the list of transactions, among others. The block size reflects the capacity of the entire block and the number of transactions it can carry. The initial block of the Blockchain is called a genesis block with a block hash of *δ*_0_. As shown in Figure 2, blocks *B*_0_ *, B*_1_ *, … B _N_* are the data blocks forming a Blockchain. ^7, 20^ Thus, a block in a Blockchain contains multiple transactions, and a transaction contains the data values involved in the transaction. ^7^

The power of a Blockchain is born out of the feature that if any data in a block is to be changed, then every subsequent data block should be modified accordingly because of the change in the block hash. This property aids in ensuring the integrity of the data blocks if the cryptographic reference to the recent block is known or agreed upon. ^21^ For instance, say the block hash of the recent block *B _N_* is known to be *δ _n_.* If data in block B1 is altered, then its block hash is altered, which eventually alters the block hash of *B _N_.* Hence, Blockchain securely stores data in a manner that prevents alteration once recorded unless there is unanimous agreement among all parties involved. ^22^ In other words, the property of data immutability is achieved through a consensus mechanism that ensures all transactions are verified and added to the Blockchain transparently and irreversibly. The result is Blockchain provides a trustworthy ledger where data integrity and transaction history are preserved, fostering transparency and reliability in various applications such as supply chain management, financial transactions and voting systems (i.e., the approval of above 50% of the network nodes is required). ^23^ De Filippi and Hassan ^23^ considers Blockchain as a decentralized database (or state machine) that considers cryptographic primitives for the surety of data integrity and authenticity. It has also been known as electronic and decentralized sometimes as immutable transaction ledger that provides cryptographic verification. These definitions underscore the fundamental aspects of Blockchain technology, emphasizing its decentralized nature, cryptographic security measures, and the immutability of transaction records. ^20^

### 2.3 Blockchain Network

One of the important aspects of a Blockchain ledger is about what makes a valid data block and who can add a new data block. A mechanism to add only a verified data block to the chain can aid in maintaining the perpetual integrity of the data. ^21^ This security feature is provided by sharing the Blockchain among a special group of computing nodes known as a Blockchain network. The broadcast network of computing nodes is known as a Blockchain network (BCN) ^21^ it has capability to maintain a copy of a synchronized storage defined as Blockchain. Each node in a Blockchain network runs a protocol for maintaining the data consistency/integrity, verify a transaction before it is added to the Blockchain, maintain operation transparency, and maintain privacy through anonymity. A Blockchain network protocol permits anyone to propagate transactions through the network for verification and to be recorded in the ledger. Some special nodes, known as miners, are responsible for verifying and then adding well-formed transactions to the ledger. The miners in the network use consensus algorithms, such as Proof of Work (PoW), and Proof of State (PoS), to agree upon the block to be added to the Blockchain. ^20, 24^ Since multiple verifiers verify the validity of a transaction, Blockchain technology (Blockchain and Blockchain network) offers decentralization and disinter-mediation of transactions between users internationally. ^25^ In brief, Blockchain technology provides a trustworthy ledger where data integrity and transaction history are preserved, fostering transparency and reliability in various applications such as financial transactions, supply chain management, and voting systems (i.e., the approval of above 50% of the network nodes is required). ^23^

**Figure 3.**
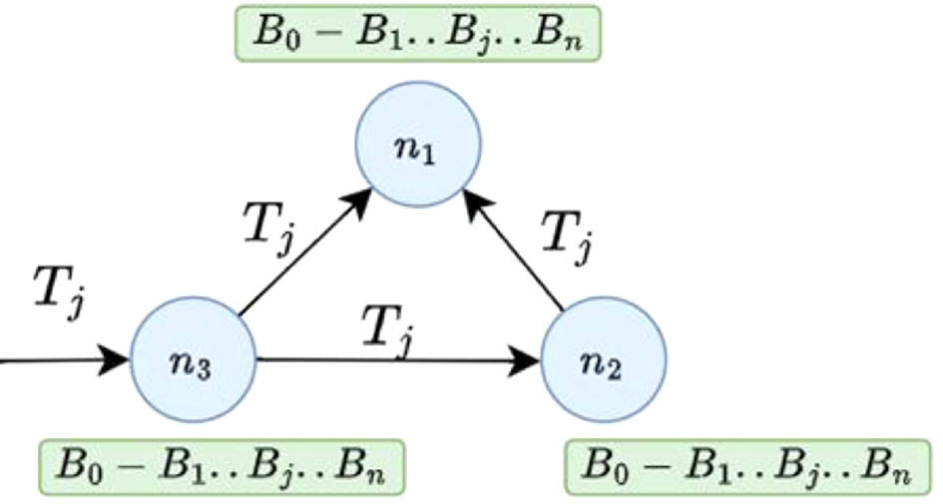
A simple network of blockchain network with three nodes. ^19^ This figure has been reproduced with permission from Adhikari et al. (2023). ^19^

Figure 3 A simple Blockchain network with three network nodes *n*_1_, *n*_2_, and *n*_3_. Each node in a Blockchain network maintains a copy of the underlying Blockchain ledger *B*_0_ *← B*_1_ *← … ← B _j_.. ← B _n_.* If a transaction *T _j_* is sent to node n3 for validation and to be recorded in the existing Blockchain, it is broadcast to connected nodes, which will also verify its correctness. ^19^ Many authors ^20, 24^ agree that Blockchain allows anyone in the world to send or participate in transactions, providing greater assurance that they are engaging with the rightful owner. This immutability is achieved through a consensus mechanism that ensures that all transactions are verified and added to the Blockchain transparently and irreversibly.

## 3. Related work

The first system of electronic voting was conceptualized by “David Shaum” in the early eighties, where public-key cryptography was utilized to ensure that electors were selected anonymously and to separate the voters’ identity from the ballots. ^26^ Estonia has adopted the electronic voting system for its national election in 2005. ^27, 28^ However, Denmark’s Liberal Alliance in 2014, started planning about integrating the Blockchain to enhance the privacy and security of national elections in Denmark. ^7^

During the later months of 2018, South Korea’s National Election Commission introduced a trial of a Blockchain-based online voting system for the South Korean elections aiming to enhance the reliability and security of voting system. As a cutting-edge innovation rooted in robust cryptographic principles, Blockchain technology facilitates easier data access for both candidates and observers, contributing to greater transparency and trust in the electoral process. However, they believe that applying Blockchain-based voting will raise the rate of buying votes. As explained in the previous section, Blockchain technology can enable the validation of whether a bribed voter adhered to their part of the deal or not. ^29^ Overall, the trial of using Blockchain-based technology in electronic voting has been successful.

This foundation empowers Blockchain-based applications to capitalize on advanced cryptographic techniques, thereby enabling them to implement highly adaptable security solutions. By leveraging these cryptographic abilities, Blockchain not only enhances the security of data and transactions but also ensures transparency and integrity across decentralized networks. ^1^ Nearly all discussions about Blockchain converge on a shared concept, though articulated with variations.

The experts have belief that the traditional voting system has problems like security of the votes hence the Blockchain is the perfect solution to make elections secure hence technologist consider it ideal platform for global democratic systems. ^12^ Blockchain technology in the electronic voting system protects the secrecy of the ballots and is ubiquitous. Further, the free and open source peer review software, allow a free and independent audit of the result, and decrease the level of confidence required from the organization or the election entity. ^30^

The distributed ledger technology allows multiple points to have the ledger instead of restricting to one location, this means that all points can have the same results unlike in traditional ballot boxes. ^12^ Therefore, failure is rare because it is decentralized and there are rare chances of being corrupted. There are different transactions where in transactions they have blocks holding previous electors transactions to ensure the voting validity, security, and connectivity. If any of the blocks are compromised due to a lack of connection between the blocks, it will be easy to detect. Real voting takes place in Blockchain. The votes by voters get sent to one of Blockchain’s nodes in the voting system, which then adds the vote to the Blockchain. The voting system has a node in each area to ensure the decentralization of the democratic framework. ^7^ Blockchain technology safeguards election records from illegal access, altering, and changing of ballots. ^1^ The consensus algorithm makes the Blockchain dominant over other technologies and hence it has increased the adaptability of the Blockchain as it can operate tasks faster, more accurately, and more efficiently than bureaucratic systems based on paper voting. ^12, 31^

As technology advanced, many countries began to integrate blockchain technology into their voting systems to enhance transparency, privacy, and accessibility in the voting process. ^32, 33^ Furthermore, many countries are adopting blockchain technology in their e-government systems. ^34^ However, the integrity of the voting process hinges on a consortium of trusted validators who are tasked with either approving or rejecting transactions. A recent study by Ref. 35 proposed an innovative adjustable Blockchain that can enhance the voting procedure, the voting mechanism, and address security concerns. Their proposed framework offers an enhanced and more secure electronic voting process, ensuring data integrity through advanced hashing technology. Blockchain technology facilitates easier data access for both candidates and observers, contributing to greater transparency and trust in the electoral process. ^29^

Blockchain technology is increasingly recognized as a cutting-edge innovation rooted in robust cryptographic principles. This foundation empowers Blockchain-based applications to capitalize on advanced cryptographic techniques, thereby enabling them to implement highly adaptable security solutions. By leveraging these cryptographic abilities, Blockchain not only enhances the security of data and transactions but also ensures transparency and integrity across decentralized networks. ^36^ Nearly all discussions about Blockchain converge on a shared concept, though articulated with variations. According to the paper by De Filippi and Hassan, ^23^ it is described as a “decentralized database (or state machine) that relies on a set of cryptographic primitives to ensure data integrity and authenticity.” Blockchain is further defined as an electronic, decentralized, immutable transaction ledger that provides cryptographic verification. These definitions underscore the fundamental aspects of Blockchain technology, emphasizing its decentralized nature, cryptographic security measures, and the immutability of transaction records. ^20^

Many authors agree that ^20, 24^ Blockchain allows anyone in the world to send or participate in transactions, providing greater assurance that they are engaging with the rightful owner. The Blockchain securely stores data in a manner that prevents alteration once recorded, unless there is unanimous agreement among all parties involved. ^22^ This immutability is achieved through a consensus mechanism that ensures all transactions are verified and added to the Blockchain transparently and irreversibly. As a result, Blockchain provides a trustworthy ledger where data integrity and transaction history are preserved, fostering transparency and reliability in various applications such as financial transactions, supply chain management, and voting systems. ^23^ Blockchain technology offers decentralization and disinter-mediation of transactions between users internationally. ^25^ For a voting system utilizing blockchain, real voting takes place in a Blockchain network. The votes by voters get sent to one of Blockchain’s nodes on the voting system, which then adds the vote to the Blockchain. The voting system has a node in each area to ensure the decentralization of the democratic framework. ^7^ Blockchain technology safeguards election records from illegal access, altering, and changing of ballots. ^1^ This technology remains prominent due to the consensus algorithm that makes it difficult to vote transactions and makes it difficult to alter or forge. ^31^

Using immutable storage, you can securely manage and maintain outsourced computational ledgers, protecting them against tampering and unauthorized access. ^37^ The work integrates Blockchain and Machine Learning for secure data transmission, employs NuCypher proxy re-encryption for efficient neighborhood encryption without cipher conversion, and utilizes ANN to optimize data delivery and record management in smart cities. ^38^A blockchain framework enabled by Hyperledger Sawtooth, offering a secure and trusted execution environment where service delivery mechanisms and protocols ensure immutable ledger storage security, along with peer-to-peer network communication for both on-chain and off-chain industrial activities. ^39^ This study proposes a lightweight Plenum consensus algorithm (BLPCA) for consortium blockchains on Hyperledger Indy, leveraging optimized Byzantine Fault Tolerance to efficiently manage large-scale decentralized socioeconomic tax traffic within hierarchical systems. ^40^ The study highlights the benefits of a permissioned private blockchain, addresses the challenges of the Proof-of- Elapsed Time (PoET) consensus mechanism in distributed applications, and proposes a lightweight middleware consensus called “B-LPoET” to improve blockchain adaptability for private chains. ^41^ In various domains Blockchain played critical role hence it has potential in voting system. Although there are limitation in the traditional voting systems that needs to be addressed by the secure voting system based on Blockchain.

## 4. Methods

This research study adopts a quantitative approach and employs an experimental design methodology, using open-source software to operate the voting systems with synthetic population data. The dataset includes citizens’ National IDs along with basic demographic information and is publicly accessible through the provided URL. ^1^

Oman’s electronic voting system is structured around diverse system models and innovative methods aimed at enhancing the efficiency, transparency, and accessibility of electoral processes across the nation. These models encompass centralized, decentralized, and hybrid approaches, each tailored to address specific requirements and challenges within Oman’s electoral system. ^42^ In the centralized model, a single authority oversees all aspects of the electronic voting process, ensuring uniformity and centralized control over voter registration, ballot distribution, and result aggregation. ^43^ The proposed model emphasizes centralized security measures and operational oversight to safeguard against potential threats and ensure the integrity of the electoral outcome. Conversely, decentralized systems distribute authority and processing power across multiple nodes or centers within Oman. This approach enhances resilience against localized disruptions or attacks, as voting operations can continue independently across different regions. ^44^ Decentralized models, such as Blockchain, often require robust coordination mechanisms to synchronize voter data and ensure consistent standards of security and accuracy throughout the electoral process. Addressing these challenges is crucial for improving the efficiency, security, and inclusivity of elections, with Blockchain technology offering potential solutions to many of these issues. ^45^ Hybrid systems in Oman blend elements of both centralized and decentralized models, offering flexibility and redundancy while maintaining centralized oversight over critical electoral functions. This model allows for adaptive responses to varying operational conditions and enhances the overall reliability and resilience of the voting system. Key methods employed in Oman’s electronic voting system include advanced biometric authentication technologies to verify voter identities securely. Biometric methods such as fingerprint scanning or facial recognition help mitigate risks of fraud and ensure that each vote is cast by the rightful individual, enhancing overall confidence in the electoral process.

The proposed model based on blockchain technology plays a pivotal role in Oman’s electronic voting system by providing a secure and transparent method of recording and verifying votes. The system helps to ensure their security and reliability as existing e-voting systems lack a clear protocol, making it difficult to ensure their security and reliability. The system utilizes the Blockchain’s distributed ledger to securely record and verify each vote. Unlike traditional centralized voting systems where trust is placed in a single authority, Blockchain-based voting ensures that all transactions (such as voter registration, voter validation, voting, and counting among others) are transparently recorded and cannot be altered retroactively. Each vote is cryptographically secured, linked to previous transactions, and distributed across a network of computers (nodes), making it extremely difficult for malicious actors to manipulate or manipulate voting data. Blockchain’s decentralized ledger ensures the immutability and transparency of voting records, making them highly resistant to tampering or manipulation and eliminates the need for intermediaries and central authorities, fostering trust among participants and reducing the risks of fraud or coercion. This aspect is particularly valuable in participatory voting systems, where ensuring the integrity of each vote is paramount to maintaining the democratic process’s legitimacy. The decentralized storage of voting data ensures the integrity and confidentiality of voting data, and Oman’s electronic voting system employs robust encryption techniques and stringent security protocols. These measures protect sensitive information from unauthorized access or manipulation, maintaining the privacy and trustworthiness of the electoral process. In addition, audit mechanisms allow for the verification and scrutiny of all actions taken during the voting process, ensuring adherence to established protocols and regulations. The use case model of the system further explains the concepts and key actors of the system that make it successful. ^12^^,^^30^

### 4.1 Use case model

**Figure 4.**
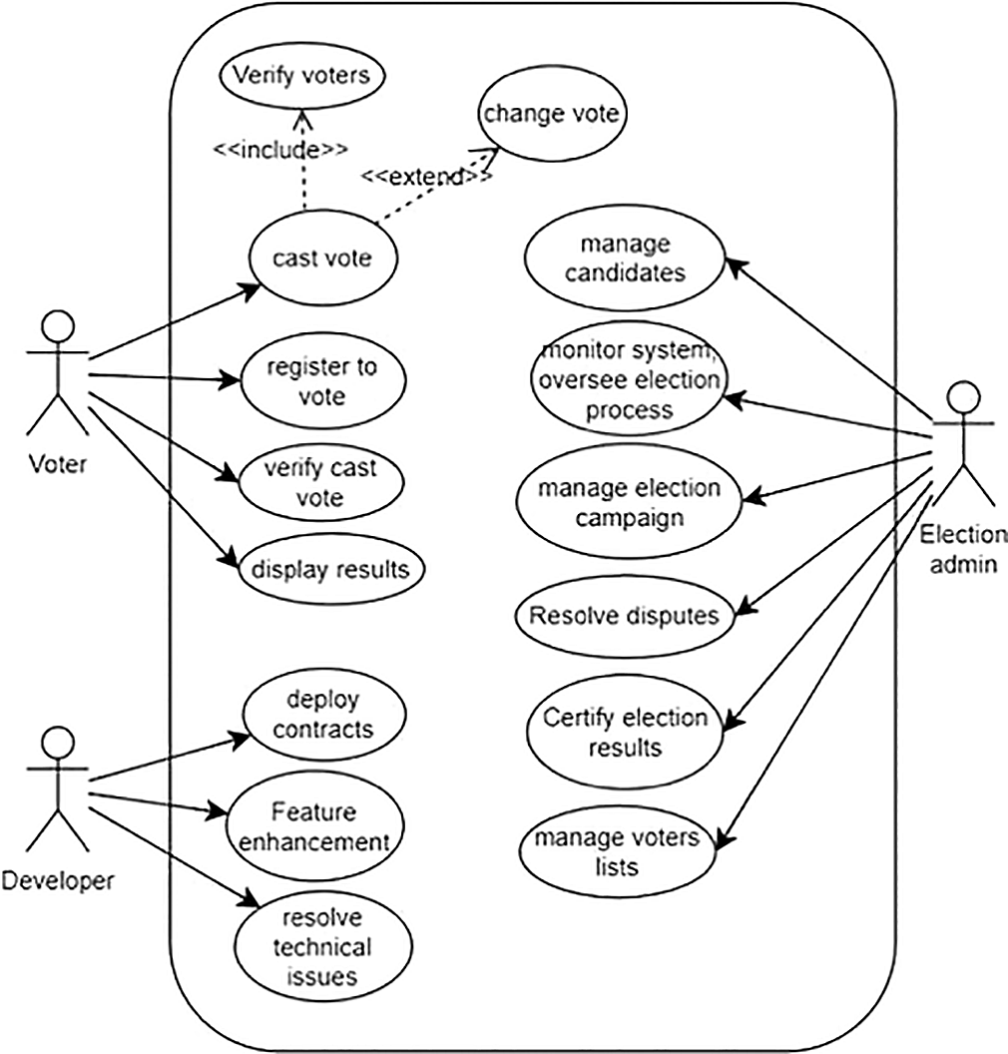
Use case model of the system.

The proposed Blockchain-based voting system involves key actors: election administrators, voters, election auditors, Ethereum nodes, and smart contract developers. Election administrators manage the process, voters cast secure electronic votes, auditors ensure integrity, Ethereum nodes maintain the network, and developers create smart contracts. Figure 4 illustrates a simplified use-case model of the system, depicting interactions among these actors and the Blockchain infrastructure, enhancing transparency and security in electoral processes.

**Election Administrators** The main operations of election administrators are as follows:

• **Manage Election:** Administrators can create, configure, and manage election campaigns. This includes setting up the parameters such as voting dates, eligible candidates, and voter eligibility criteria.
• **Monitor System:** Administrators can monitor the overall system health, including Blockchain network status, transaction verification, costs, and any potential security threats.
• **Generate Reports:** Administrators may need to generate various reports related to election results, voter turnout, and system performance for auditing and analysis purposes.
• **Oversee Election Process:** Election authorities can oversee the entire election process, ensuring fairness, transparency, and compliance with electoral regulations.
• **Resolve Disputes:** Election authorities can intervene and resolve disputes or challenges related to voter eligibility, ballot integrity, or other election-related issues.
• **Certify Election Results:** Election authorities can certify the final election results based on the data recorded on the Blockchain ledger, providing official validation of the outcome.

**Voters** Voters are the general public illegible for a voting campaign. The main operations of voters are as follows:

• **Register to Vote:** Voters can register themselves on the Blockchain-based system to participate in elections. This process may involve identity verification and confirmation of eligibility criteria. To simplify the process, voters can register to the Blockchain system for a voting campaign via an intermediate web application server operated by a government body such as the election commission.
• **Cast Vote:** Voters can securely cast their votes for their chosen candidates. The system should ensure anonymity, integrity, and verification of each vote.
• **Verify Vote:** Voters can verify that their vote has been correctly recorded on the Blockchain ledger, providing transparency and confidence in the voting process.
• **View Candidate’s details:** Voters can access information about candidates, their agendas, and other relevant details to make informed voting decisions.

**Auditors**

• **Audit System Integrity:** Auditors can verify the integrity of the smart contracts, and voter registration, among others ensuring that the system operates without any manipulation or fraudulent activities.
• **Review Transaction History:** Auditors can review the transaction history stored on the Blockchain ledger to validate the accuracy and transparency of the voting process.
• **Verify Compliance:** Auditors can ensure the system complies with relevant regulations and standards governing elections, data privacy, and security.

**Web Application and Smart-Contract Developers**

• **Develop Smart Contracts:** Developers can create and deploy smart contracts that govern the rules and logic of the voting system, ensuring transparency, security, and automation of voting processes.
• **Enhance System Features:** Developers can propose and implement enhancements to the Blockchain-based voting system to improve scalability, usability, and security.
• **Resolve Technical Issues:** Developers can troubleshoot and resolve technical issues within the system, such as bugs, performance bottlenecks, or vulnerabilities.

**Ethereum Nodes** Ethereum nodes play a prominent role in the Blockchain-based voting system by actively listening for and processing critical transactions such as voter verification, vote casting, and election result dissemination. Their active participation is integral to maintaining the integrity and transparency of the electoral process. Therefore, Ethereum nodes are considered principal actors within our system, ensuring secure and decentralized management of voting operations. This collaborative effort among nodes strengthens the reliability and resilience of the voting system, bolstering confidence in the accuracy and fairness of election outcomes.

• **Verify voters:** In Ethereum-based voting systems, nodes verify voter identities before they can cast their votes. Nodes maintain the Blockchain’s integrity by executing smart contracts that authenticate voters and record their votes securely. This decentralized approach ensures transparency and safeguards against manipulation, promoting trust in the electoral process.
• **Receive cast votes:** In Ethereum-based voting systems, Ethereum nodes receive and validate votes cast by voters for candidates, ensuring the integrity and transparency of the voting process through decentralized verification and Blockchain recording.
• **Display/Print results:** In Ethereum-based voting systems, Ethereum nodes validate votes, tally election results, and securely record the outcome on the Blockchain, ensuring transparency and immutability in the electoral process.

**System Architecture** The architecture of a simplified Blockchain-based distributed voting system comprises four tiers: the front-end tier (Tier-4) provides user interfaces for voter interactions, the Web2 application services tier (Tier-3) manages business logic and user authentication, the Web3 Blockchain network smart contract services tier (Tier-2) hosts smart contracts governing the voting process, and the storage services tier (Tier-1) ensures secure storage of encrypted voter data and transaction records, collectively enhancing transparency, security, and reliability in electoral operations.

**Tier-1**

**Figure 5.**
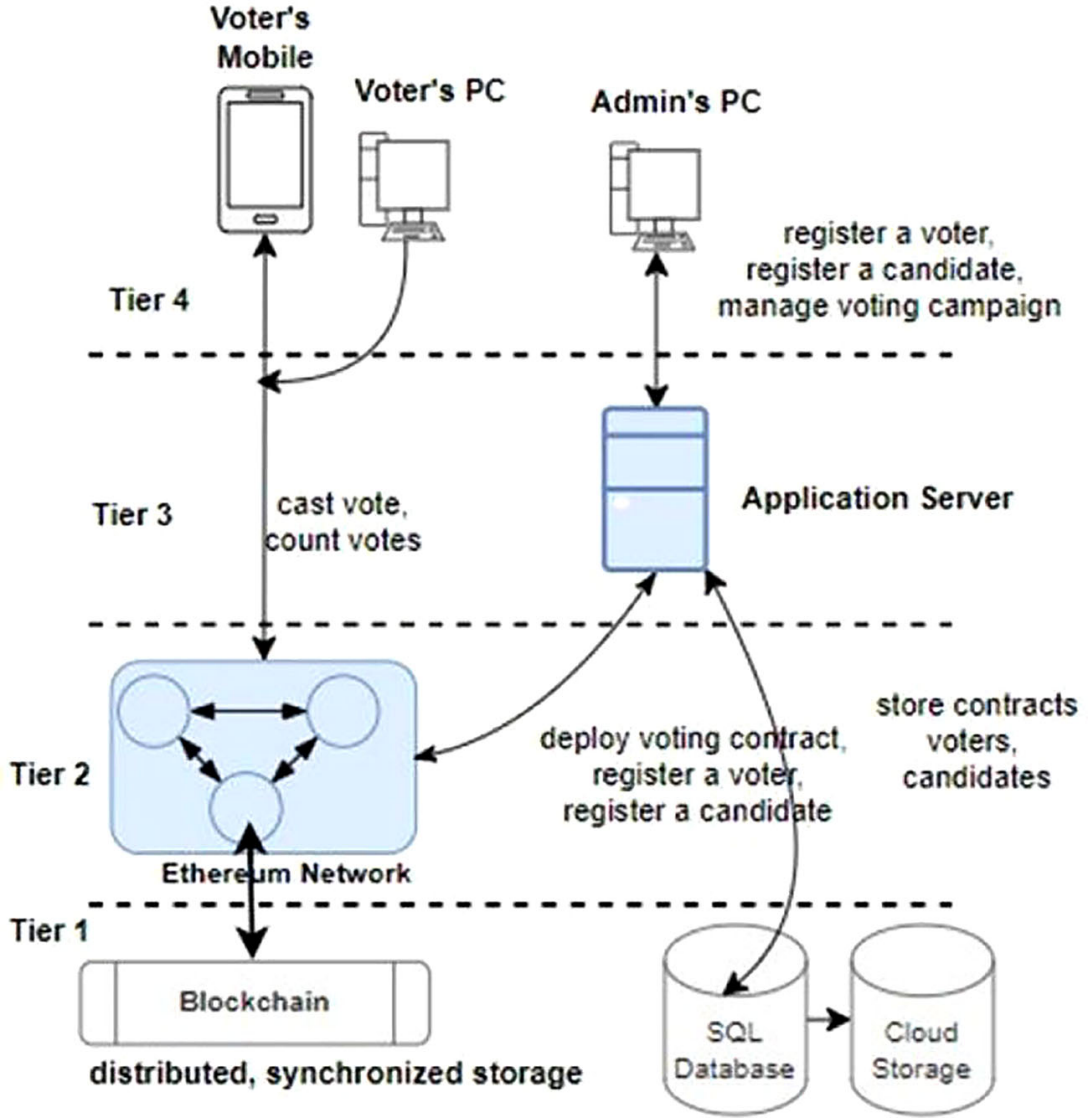
A simplified Blockchain-based distributed voting system architecture.

As illustrated in Figure 5, tier-1 encompasses critical data storage services like MySQL for traditional data management and Blockchain ledger for immutable, decentralized record-keeping. Tier-3 voting application server utilizes MySQL-based storage engines to efficiently store and retrieve user credentials, voter information, and administrative data. Meanwhile, the Ethereum network in tier-2 involves Ethereum nodes maintaining individual copies of the Blockchain ledger that guarantees transparency, security, and integrity in the voting process by securely recording each vote as a tamper-proof transaction. This Tier-1 layer forms the foundation of the application’s architecture, combining the reliability of traditional databases with the innovation and trust of Blockchain technology to uphold the integrity of the voting process. In other words, a Blockchain ledger ensures that a voter can only cast a single vote for a candidate by restricting the backtracking of the cast votes to the candidates and producing trustworthy results of a voting campaign.

**Tier-2**

Tier 2 is the critical service layer of a trust-less distributed voting system. It is composed of an Ethereum Blockchain network. An Ethereum network consists of thousands of computing nodes that individually store a copy of a distributed, synchronized database known as Blockchain [citation]. The network is a distributed, peer-to-peer network. ^46^ The major feature of such a network is that each Ethereum node can store smart contracts in the Blockchain and run them in its Ethereum virtual machine. A smart contract is a stored procedure that performs atomic digital transactions, the outcome of which can be verified by the participating Ethereum nodes and committed in the Blockchain database. In a Blockchain network such as Ethereum, every transaction is stored in the Blockchain, and such storage is immutable and temper-proof. ^47^

The Ethereum network provides the following services to our voting system:

1. Register voters and store them in the Blockchain.
2. Register and store election officers and election administrator mandates in the Blockchain.
3. Register a valid vote cast by a registered voter for a candidate
4. Respond to the user’s query to check if a voter is registered.
5. Respond to the user’s query to check the count of votes received by a candidate.

The operations perform all of the operations in the smart contract deployed in theEthereum network.

**Tier-3**

Tier 3 is composed of the web application services that can be utilized by election administrators for the following tasks:

1. Register the voting campaign officers.
2. Register the voters for the voting.
3. Register the candidates for the voting campaign.
4. Deploy the voting campaign smart contracts to the Ethereum Blockchain.

Tier-3 application servers utilize Tier-1 relational database services such as MySQL for data storage. Our tier-3 web application service was built in C#. Alternatively, we can also use cloud storage services such as database.com, Oracle, Amazon Aurora, IBM Db2, and BigQuery. ^48^

**Tier-4**

Tier 4 of the architecture encompasses a diverse array of end-user devices, including smartphones, personal computers, tablets, and client applications such as web browsers and crypto-wallets. These devices play a pivotal role in the Blockchain- based voting system by serving as interfaces through which stakeholders, including voters and election administrators, interact with the electoral process. One of the distinctive features of devices in this layer is their ability to securely host and manage crypto wallets. Crypto wallets store cryptographic keys that authenticate users and authorize their actions within the voting system. For voters, these wallets enable secure and anonymous casting of votes, ensuring confidentiality and integrity throughout the voting process.

Election administrators utilize crypto wallets to manage voter registration, monitor election activities, and validate results, maintaining transparency and accountability in electoral operations. Moreover, end-user devices in tier 4 facilitate seamless connectivity to the Web3 Blockchain network, where smart contracts execute and enforce the rules governing the voting process. This direct interaction with the Blockchain ensures that all transactions, from voter registrations to ballot submissions and result tabulations, are recorded immutably and transparently on the distributed ledger. By leveraging the capabilities of smartphones, computers, and other client applications, the voting system enhances accessibility for voters, enabling them to participate conveniently from any location with internet access. This inclusivity fosters higher voter turnout and engagement in democratic processes, while the secure handling of crypto wallets and Blockchain interactions ensures the system’s resilience against cyber threats and fraud. The voters utilize the client applications for the following tasks:

1. To cast the votes for the candidates.
2. To register for the voting campaign.

We created programs based on .Net Core 7 to simulate the operations of the tier-4 devices and applications. However, for production purposes, any browser-based client applications can be developed to support multiple operating systems such as Windows, MacOS, Linux, Android, iOS, among others.

## 5. Experimentation setup

We developed a simulation package in the *C*# .Net platform to determine the proposed system’s efficiency. The smart contract was developed in *C*# and deployed in the Ethereum-based Geth test chain provided by Nethereum. The Blockchain network Geth is deployed on a machine with Microsoft Windows 10 Enterprise OS, a 12th Gen Intel ^®^ Core™ Intel i7-12700, 2100 Mhz, 12 Cores processor, and 16GB installed Physical Memory (RAM).

We set up a testbed based on the Geth test chain and .Net Core Clients using Nethereum—the .Net integration library to connect to Ethereum ^2^. The library contains APIs that simplify the access and smart contract integration with Ethereum nodes. In the context of communication design, the protocol is HTTP, which can be switched to HTTPS for a secure channel. However, for communication simplicity, we let the protocol be HTTP-based. We implemented the algorithms as listed in this section in *C*#.Net.

Algorithm 1. **SmartContract *π.***

1: **Struct:** Election {uint eid; uint year; string name; uint type}

2: **Storage:**

3:   address ContractOwner

4:   Elections *←* MAP (address => Election);

5:   Voters *←* MAP (address => uint);

6:   Candidates *←* MAP (address => uint);

7:   Voters *←* MAP (address => uint);

8: **procedure** CandidateRegistration (address ID)

9: **if** Candidates [ID] **then**

10:   return FALSE

11: **end if**

12: **if** ContractOwner == Message. Sender **then**

13:   return FALSE

14: **end if**

15:   Candidates [ID] = TRUE

16: **end-procedure
**

17: **procedure** VotersRegistration (address ID)

18: **if** Voters [ID] **then**

19:   return FALSE

20: **end if**

21: **if** ContractOwner == Message. Sender **then**

22:   return FALSE

23: **end if**

24:   Voters [ID] = TRUE

25: **end-procedure
**

26: **procedure** CastVote (address ID)

27: **if** Voters [ID] == TRUE **then**

28:   return FALSE/*voter already voted*/

29: **end if**

30: **if** Candidates [ID] **then**

31:   return FALSE/*candidate is registered.*/

32: **end if**

33:   Candidates [ID] += 1

34:   return TRUE

35: **end-procedure
**

36: **procedure** VoteCount (address ID)

37: **if** Candidates [ID] **then**

38:   return Candidates [ID]

39: **end if**

40:   return FALSE

41: **end-procedure
**

42: **procedure** Constructor()

43:   ContactOwner = Message.Sender

44:   return TRUE

45: **end-procedure
**

Algorithm 2. **DeploySmartContract.**

1: **Inputs:** EthereumNetwork *chi ←* ( *networkid* = *{}, url* = *{}*), SmartContract *π*/*Hereafter, (..) is a tuple defining the object*/

     /*{..} is a place-holder for appropriate values.*/

2: **Outputs:** SContractReceipt *r ← (time, contract-address, message)*

3: *v.*connect_to( *χ*); /*connect the the blockchain network network χ*/

4: *r ← χ.*deploy( *π*); /*execute a transaction to deploy the smart contract *π* on the blockchain network χ*/

Algorithm 3. **CandidateRegistration.**

1: **Inputs:** EthereumNetwork *χ*, SmartContract *π*

2: **Outputs:** CRegistrationReceipt *r ← (time, address, message)*

3: **Initialize candidates** *C ←* { *c*_0_, *c*_1_, .. *c _n_*}; *c _i_ ← (ID, name, SSN, dob, election-id)*

4: **for** *c* in *C* **do**:

5: *c.*connect_to( *χ, π*);

6: *r ← c.*cregister(); /*execute a transaction to register a candidate for an election with ID, eid.*/

7: **end for**

Algorithm 4. **VotersRegistration.**

1: **Inputs:** EthereumNetwork *χ*, SmartContract *π*

2: **Outputs:** RegistrationReceipt *r ← (time, address, message)*

3: **Initialize voters** *V ←* { *v*_0_, *v*_1_, .. *v _n_*}; *v _i_ ← (ID, name, SSN, dob, election-id)*

4: **for** *v* in *V* **do**:

5: *v.*connect_to( *χ, π*); /*establish the connection*/

                             /* to the network and the smart contract*/

6: *r ← v.*register(); /*execute a transaction to register a voter for an election with ID, eid.*/

7: **end for**

Algorithm 5. **CastVote.**

1: **Inputs:** EthereumNetwork *χ*, SmartContract *π*, Voters *V*, Candidates *C*

2: **Outputs:** VoteCastReceipt *r ← (time, address, message)*

3: **for** *v* in *V* **do**:

4: *v.*connect_to( *χ, π*);

5: *r ← v.*cast_voteto( *c*: *c ∈ C*); /*voter *v* voting the candidate *c ^′^* */

6: **end for**

## 6. Results and Discussion

As part of the prototyping, the following tasks were completed:

1. Load a 1000 list of voter-related data to SQL servers. Each voter is identified by a synthetic Social Security Number (SSN) and has other information such as full name, address, phone number, and email address.
2. A client program developed in *C*# .Net produced 1000 voting (casting votes) transactions encompassing 1000 total voters identified with synthetic Social Security Numbers (SSN) executed against the network.

**Figure 6.**
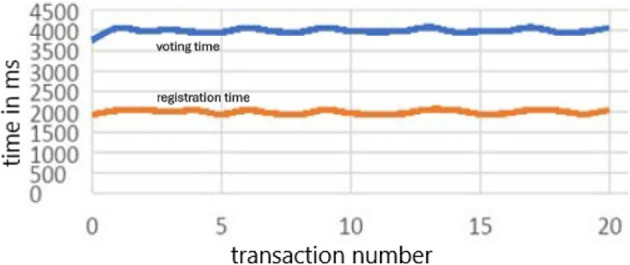
Transactions Times in Millisecond for registration and voting transaction.

Two main transactions, voter registration and vote casting, were executed in two different ways:

1. Synchronous transactions: This method synchronously submits transactions to the Blockchain network. In other words, individual voter registration and individual vote-casting transactions were executed one after another. A total of 1000 transactions for each were executed to measure the execution time. Figure 6 shows the time required for registration and voting 20 individual transactions run synchronously.
2. Asynchronous transactions: This method submits transactions to the Blockchain network asynchronously. The voter registration and vote-casting transactions were executed asynchronously in batch sizes of 10, 20, 30, and 50. For example, with a batch size of 10, 10 voter registration transactions and 10 vote-casting transactions were asynchronous. The final vote registration and vote casting time for each batch were measured. Figure A shows the time required to execute 1000 transactions for registration and voting. The red plot is the line plot for the time (MS) taken by transaction for voter registration, the grey line plot is for the time (in MS) taken by transaction for voting and receipt of the confirmation, and the blue line plot is the total time taken for both transactions. The spikes in time measurements in asynchronous transactions are often a result of the dynamic and distributed nature of asynchronous systems, where various factors such as network latency, resource contention, flow control queuing delays, and resource allocation and scheduling can influence transaction processing time.

**Figure 7.**
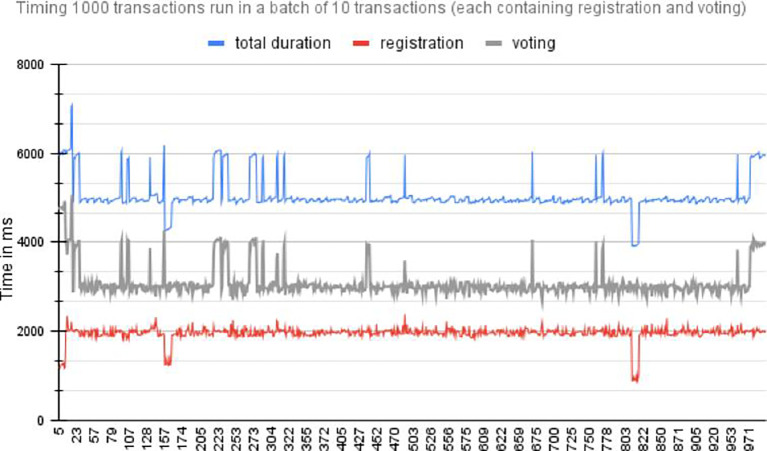
Performance per 1000 Transactions.

Figure 7 shows the time required by asynchronous execution of 100 batches, each consisting 10 transactions for voter registration and vote casting. The red plot is the line plot for time (MS) taken by transaction for vote casting and receipt of the confirmation, and the blue line plot is the total time taken for voter registration.

**Figure 8.**
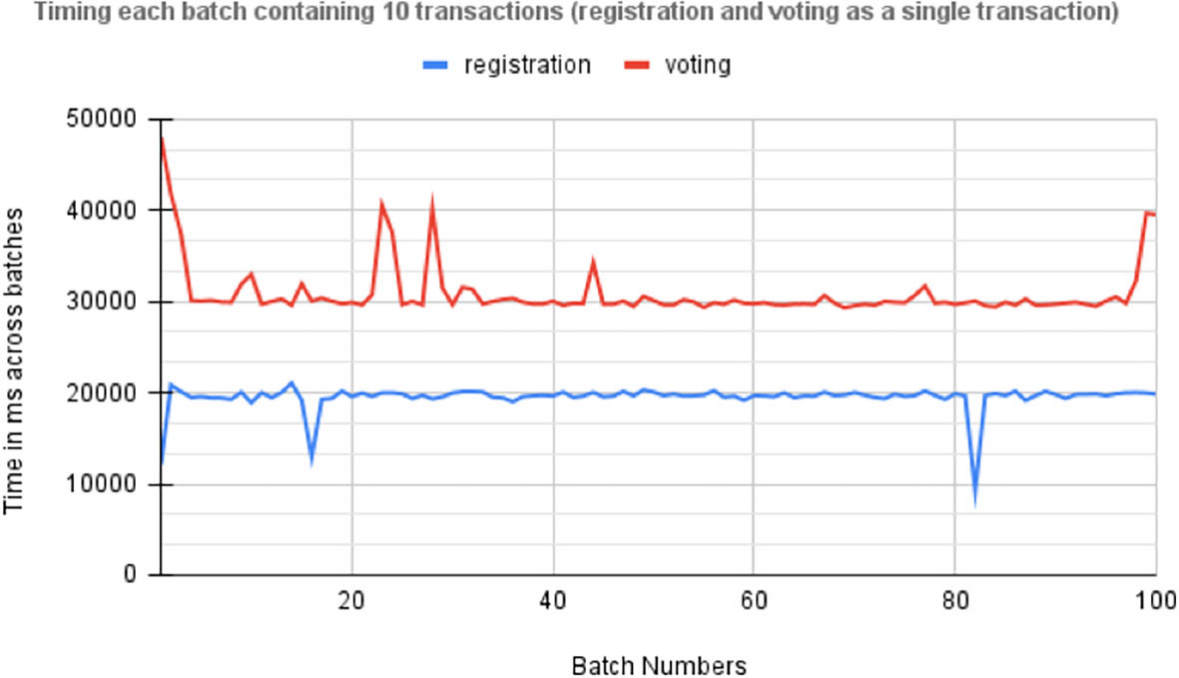
Times across batches each containing 100 transactions.

Figure 8 Timing 100 batches of transactions each containing 10 individual transactions for registration and voting.

**Figure 9.**
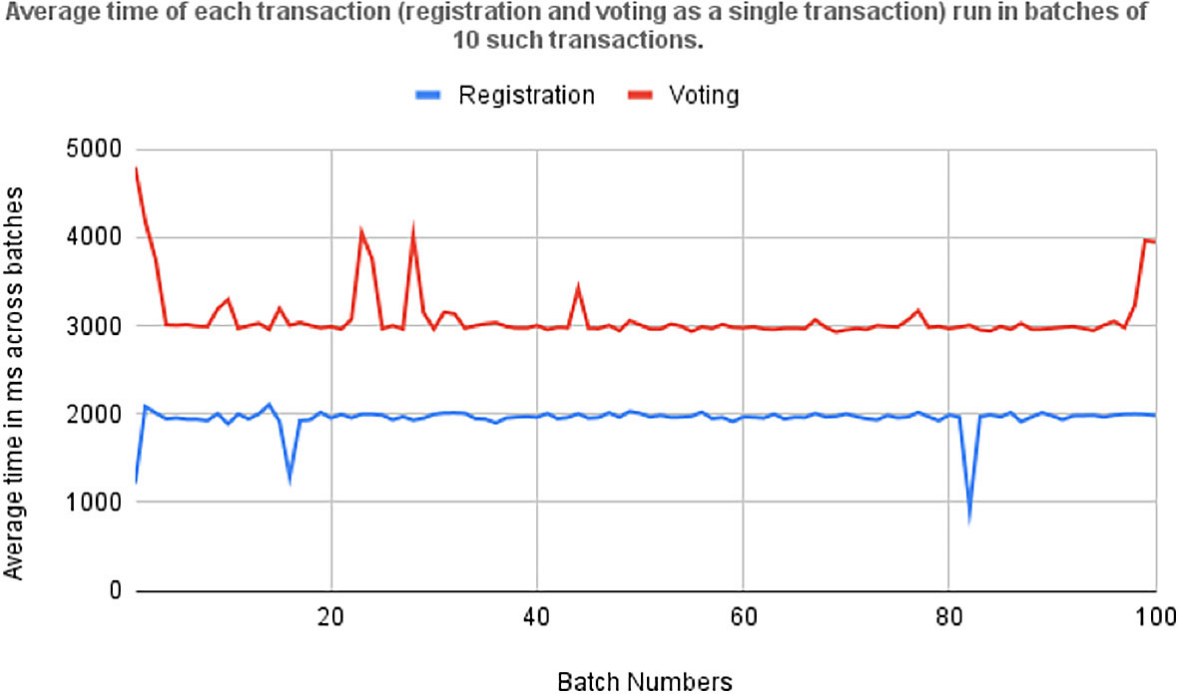
Average time required by asynchronous execution of 100 batches.

Figure 9 displays the average time required for the asynchronous execution of 100 batches, each containing 10 transactions for voter registration and vote casting. This metric is crucial for evaluating the efficiency of the Blockchain-based voting system, particularly in handling concurrent transactions during electoral peaks. It underscores the responsiveness and reliability of end-user devices like smartphones and computers in securely processing these transactions, highlighting the system’s scalability and performance under varying loads. These insights are essential for optimizing the voting system’s infrastructure to ensure robustness and effectiveness in electoral operations.

## 7. Conclusion

It has been concluded that Blockchain technology has enhanced the integrity and security of digital voting systems in Oman. By addressing critical issues such as data integrity, fraud prevention, and transparency, Blockchain can significantly bolster voter trust and participation in the electoral process. The Blockchain-based voting system not only ensures a tamper-proof and transparent voting system but also maintains voter privacy and facilitates real-time auditing. Successful implementation, however, requires overcoming several challenges, including technological readiness, scalability, and societal resistance to change. The study highlights the necessity for a robust regulatory framework and comprehensive public awareness initiatives to foster acceptance and understanding of Blockchain technology in the electoral context. Policymakers are urged to initiate pilot projects to evaluate the practical implications of Blockchain integration, ensuring a gradual and informed transition. Ultimately, the adoption of Blockchain in Oman’s electoral system could serve as a model for other nations seeking to enhance the security and credibility of their voting processes. Oman has the opportunity to set a precedent in electoral innovation, ensuring that its democratic processes are robust, transparent, and trustworthy. While the proposed model offers numerous benefits, there are risks and limitations to consider, including the need for advanced infrastructure and technical expertise, potential scalability issues as voter numbers grow, and resistance from the public and stakeholders unfamiliar with or concerned about the reliability of Blockchain technology.

## Ethics and consent

This study does not involve human participants or animals, and therefore, ethics approval was not required. No sensitive data were used, and no real personal information was collected.

## Author contributions statement

The authors hereby confirm that we all have made a substantial contribution. A.K.S. has contributed to idea generation and provided direction for the research, and designed the methodology, and wrote the original draft. However, N. A, A.S.S are engaged in the experiment and results. A. N, S. A and H. A have contributed to the partial write-up, visualized the results. All authors reviewed and approved the final version of the manuscript.

## Data Availability

Figshare: Blockchain based secure voting system, https://doi.org/10.6084/m9.figshare.28113140.v1. This project contains the following underlying data:
• Polling folder • Readme • Data file – Citizen data_with_national_id.csv The data supporting the findings of this study are available on under the license GPL 3.0+. All other relevant data are included in the manuscript.

## Footnotes

^1^

https://url.us.m.mimecastprotect.com/s/LI24CkRwomf5wBgE7T2fyTGMWiA?domain=figshare.com

^2^

https://docs.nethereum.com/en/latest/
