# Blockchain-enhanced electoral integrity: a robust model for secure digital voting systems in Oman

**DOI:** 10.12688/f1000research.160087.2

**Published:** 2025-04-28

**Authors:** Abdul Shaikh, Naresh Adhikari, Amril Nazir, Abdul Salam Shah, Saranjam Baig, Hafedh Al Shihi

**Affiliations:** 1 Information Systems, Sultan Qaboos University, Muscat, Muscat Governorate, 123, Oman; 2 Department of Computer Science, Slippery Rock University, Slippery Rock, Pennsylvania, 16057, USA; 3 Information Systems and Technology Management, Zayed University, Abu Dhabi, Abu Dhabi, 144534, United Arab Emirates; 4 School of Computer Science, Faculty of Innovation and Technology, Taylor's University, Subang Jaya, Selangor, Malaysia; 5 College of Economic and Political Science, Sultan Qaboos University, Muscat, Muscat Governorate, 123, Oman

**Keywords:** Blockchain Technology, Electoral Integrity, Digital Voting Systems, Voter Security, Transparent Elections, Secure Voter Authentication, Decentralized voting, Oman

## Abstract

**Background:**

Ensuring the security and trustworthiness of a digitized and automated electoral process remains a significant challenge in democratic systems. As digital voting systems are increasingly being investigated worldwide, ensuring the integrity of the process using robust security measures is of great importance. This paper presents a simplified model to enhance electoral integrity by leveraging Blockchain technology in the context of Oman’s digital voting system. The model uses Blockchain technology to create a secure and trustworthy voting environment, addressing key vulnerabilities in digital electoral systems.

**Methods:**

The research utilized a quantitative approach, employing an experimental design methodology using open-source software to simulate voting systems. Synthetic population data is used to operate these systems, while advanced biometric authentication technologies verify voter identities. Blockchain technology is leveraged to ensure secure vote recording, with smart contracts used to authenticate voters and securely record votes. Additionally, synchronous transactions are executed for both voter registration and voting processes, enhancing the overall security and efficiency of the system.

**Results:**

The experimental results show that Blockchain enhances electoral integrity and security in Oman’s voting system, improving elections’ transparency and reliability. The performance evaluation of the model focuses on efficiency, reliability, and scalability metrics. Asynchronous transactions are utilized to improve processing time for voter registration and voting. Election administrators can manage, monitor, and certify election results, while Ethereum nodes ensure decentralized verification and transparency in the voting process.

**Conclusion:**

This research offers insights for policymakers to consider Blockchain for electoral reforms, addressing issues like data integrity, fraud prevention, and transparency to boost voter trust. A strong regulatory framework and public awareness are crucial for successful implementation. Pilot projects are needed to assess Blockchain’s practical impact. Oman could lead global innovation in electoral technology, though infrastructure and public resistance challenges must be managed.

## 1. Introduction

Governments around the world use voting to implement democracy, as elections are the backbone of democracy that showcases the views of citizens in terms of votes. ^1^ A vote formally expresses a choice between two or more candidates or courses of action. This process typically involves using a ballot, a show of hands, or other methods to indicate one’s preference. ^2^ Voting is the foundation of any effective democracy; therefore, it must be accessible and secure for all citizens. Ensuring that every qualified individual can participate in voting process is vital to preserving the integrity and legitimacy of democratic systems. ^3^ It ensures that all perspectives are considered, strengthens equity and justice, and improves public confidence in the electoral process. ^4^ Traditional voting was often conducted by displaying thumbs-ups (approval) or thumbs-downs (disapproval) from the voters. The candidate who received the highest thumbs-up was declared the winner. This straightforward method of voting allowed for open and immediate visible feedback, making it easy to determine the outcome. ^5^ This simple approach was effective in small gatherings or assemblies where decisions needed to be made quickly and transparently. ^6^ The electoral system must have ultimate safety to ensure access to legitimate users to avoid any inside and outside interference that can prevent votes from being cast or the voter’s vote from being altered. ^7^ Bracamonte et al. assert that a successful voting system meets the electoral system criteria that optimize the system’s advantages for the citizens and the administration. ^8^

There are several challenges associated with traditional and voting methods. Firstly, security concerns are significant, as these methods are susceptible to manipulation, fraud, and cyber attacks, raising doubts about the integrity of election results. ^9^ Secondly, traditional voting lacks transparency, leading to questions about the results’ legitimacy due to opaque processes. In addition, accessibility remains an issue for certain demographics, such as individuals with disabilities or those in remote locations, who may face barriers to participating in the voting process. ^10^ In addition, the high costs associated with physical infrastructure, printed ballots, and manpower contribute to the cost of elections. In addition, manual or electronic vote-counting processes can be time-consuming, resulting in delays in announcing results. Finally, inconvenient polling locations, long waiting times, or limited voting hours can discourage voter turnout, undermining the democratic process. ^11– 13^ Addressing these challenges is crucial to improving the efficiency, security, and inclusion of elections, and blockchain technology offers potential solutions to many of these issues.

Blockchain technology comprises exchanging and storing verified transactions over a distributed network of computing nodes. The technology leverages techniques that ensure the integrity and security of data by maintaining a distributed ledger, also known as a blockchain, which is a series of interlinked data records. Once added to the blockchian, each transaction or record is linked to the previous entry using the one-way hash functions of the previous block, forming a chronological chain of blocks. ^14^ The blockchains are maintained across a network of computers, also known as qualifying nodes.

The decentralized verification of transactions before they are added to the temper-proof blockchain eliminates the need for a central authority, as all participants in the network have access to the same information, ensuring transparency and reducing the risk of fraud such as double spending. ^15^ The technology involves complex inter-process communication that verifies and validates each transaction, making it nearly impossible to alter or manipulate once the transaction is added to the blockchain. ^16^ For example, Bitcoin, created by Satoshi Nakamoto in 2008, is a popular application of blockchain technology that facilitates the payments of transactions between two parties without any intermediary, such as credit card companies and banks. ^16^

The proposed electronic voting model leverages blockchain technology to enhance security, transparency, and trust in the electoral process. This research considers the case of Oman to present and compare both traditional and electronic systems in the election process, which is reflected in the electronic voting system used in the recent Omani elections held on 27 ^th^ October 2019. This research considers the case of the electoral system in Oman to present and compare traditional and electronic electoral systems. The contributions of this research are as follows:

• Identify and discuss security and trust issues with traditional electoral systems.
• To propose and prototype a simplified blockchain-based electoral system.
• To implement and test the smart contracts required for blockchain-based electoral system.
• Evaluate the proposed digital blockchain-based electoral system regarding performance matrices such as the time required for registration and voting.

The rest of the paper is organized as Section 2 discusses the preliminaries, Section 3 presents related works published on the Blockchain and electronic voting, and Section 4 contains system models and methods used in the proposed model. Section 5 presents the experimental setup of the study while Section 6 presents a detailed discussion of the results followed by Section 7 conclusion.

## 2. Preliminaries

### 2.1 The Omani Election System: An overview

There are two main councils in Oman, Majlis Al-Shura (Lower House) and Majlis Al Dawla (Upper House). These two councils are under the name of “Majlis Oman”. Majlis Al Dawla members were appointed previously by His Majesty Sultan Qaboos and currently by His Majesty Sultan Haitham, from previous government officials and prominent citizens. On the other hand, citizens choose Majlis Al-Shura members by voting, but this is not mandatory. Majlis Al-Shura was founded in 1991, as a substitute for the traditional consultative council of the state. In Oman, there are 61 Wilayats (administrative division). Each member of the Majlis represents one Wilayat, and if the population of the Wilayat exceeds 30,000 people, two members represent that Wilayat. The membership term in Majlis Al-Shura is four years. ^17^

The Ministry of Interior (MoI) is responsible for the elections in Oman. The voter must first register at the MoI to have the right to vote. There are some conditions for voters to be eligible to vote ^18^:

• Must have Omani nationality.
• Must be 21 years old or above.
• Must vote in the voter’s Wilayat or the address on the voter’s identity card (ID).

On the day of the election, each voter casts his/her vote at the election station located in his/her Wilayat. In the election center, the verification is done manually by the administration committee stationed at the election station through the person’s ID. After the verification has been done successfully, the voter is given the voting paper that the voter chooses, marks the candidate of his/her choice, and drops the paper in the selection box. Each voter can vote only once and for a single candidate. At the end of the election, the election audit committee at each station reviews all voting ballots manually and calculates the election results. This lengthy process comes with several drawbacks, such as:

• The voter has to visit the election station physically to be able to cast a vote (which depends on the location of Wilayat).
• The results have to be calculated manually, which is time-consuming and prone to many problems, such as manipulation and missing count.
• There is a high possibility of fraud, which can be perceived while calculating the result.

**Figure 1.**
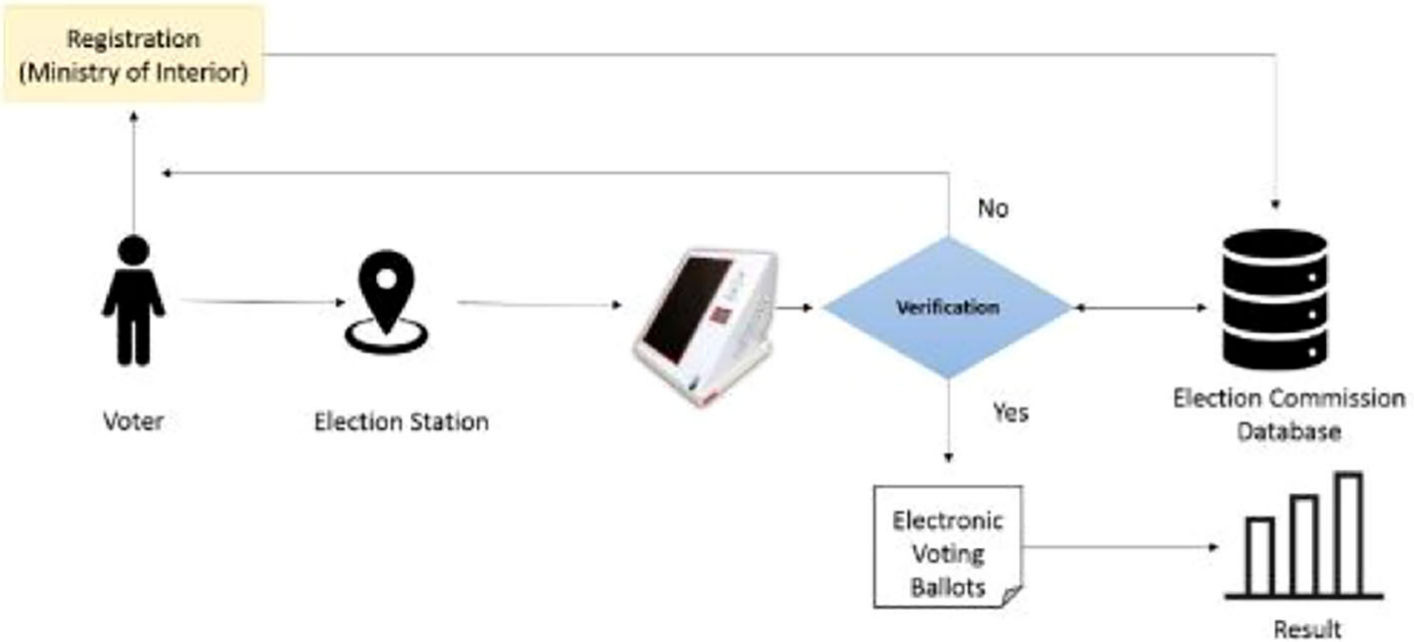
Use case model of the system.

However, traditional voting, which depended on paper and pen, no longer exists in Oman. Oman has implemented the electronic voting system to improve the electoral process by keeping it more traceable and verifiable. The recent 9 ^th^ general election in Oman, held on 27 ^th^ 2019, was conducted using an electronic voting system. The electronic election is executed through an electronic device called ‘Sawtak’ and is distributed at each election station. It allows the verification process to be automatically completed once the user enters his/her ID, after which the voter has to complete the fingerprint authentication process. If the user is verified successfully, then the electronic ballot list of the candidates is displayed for 120 seconds for selection. This list is not available to unauthorized users, such as unregistered users from the MoI and users who try to access the list from different election stations. Once the list appears and the voter picks a candidate, an electronic ticket is displayed on the device that includes the username and the chosen candidate to verify the completion of the electoral process. The researcher illustrates the election process in Oman in Figure 1.

### 2.2 Blockchain

**Figure 2.**
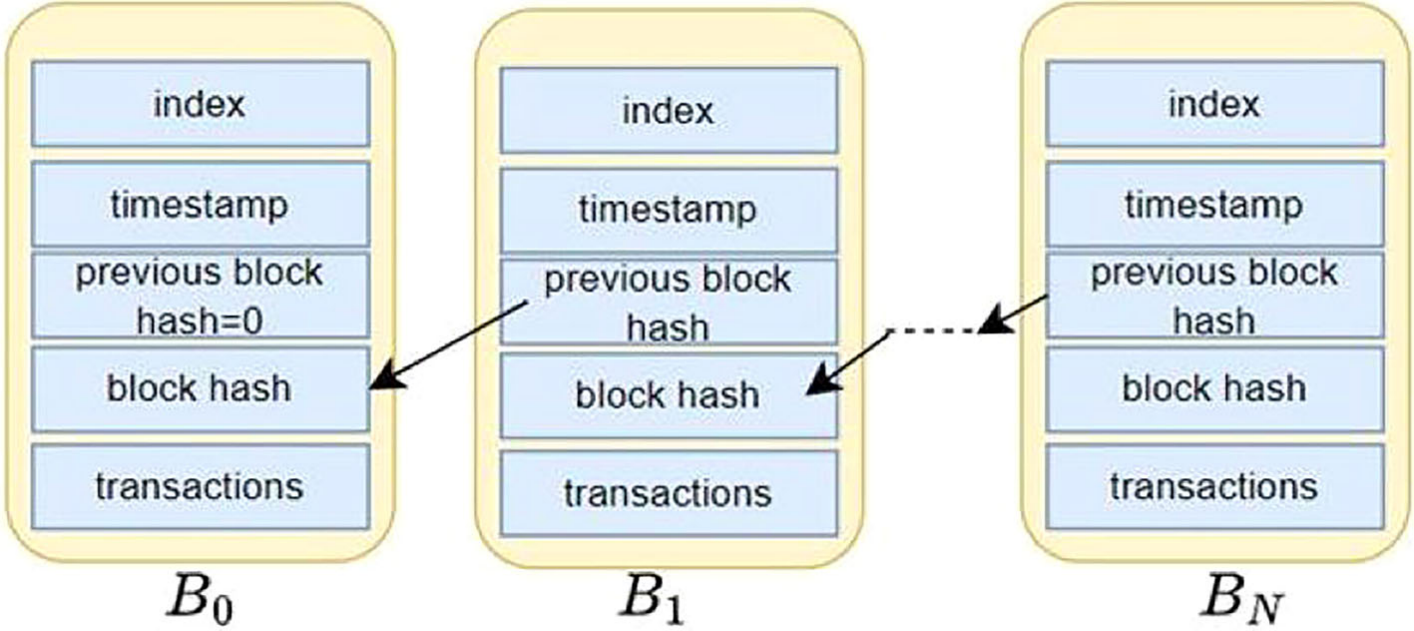
Structure of a simple Blockchain ledger. ^19^ This figure has been reproduced with permission from Adhikari et al. (2023). ^19^

Blockchain can be defined as a chain of data blocks that represent an ordered storage of the blocks. ^19^ Each data block in the chain is linked to the previous block by containing a cryptographic reference to the previous block. The cryptographic reference, also known as block hash, is usually the secure hash value of the contents in the previous block. If *M* are data contained in a block, then *δ* = *H*( *M*) is the block hash, where *H*( *.*) is a secure, one-way function such as SHA-2, SHA-3, among others. A block can contain different information such as block index, timestamp, previous block’s block hash, number of transactions in the block, and the list of transactions. The block size reflects the capacity of the entire block and the number of transactions it can carry. The initial block of the blockchain is called a genesis block with a block hash of *δ*_0_. As shown in Figure 2, blocks *B*_0_ *, B*_1_ *, … B _N_* are the data blocks forming a blockchain. ^7, 20^ Thus, a block in a blockchain contains multiple transactions, and a transaction contains the data values involved in the transaction. ^7^

The power of a blockchain is born out of the feature that if any data in a block is to be changed, then every subsequent data block should be modified accordingly because of the change in the block hash. This property aids in ensuring the integrity of the data blocks if the cryptographic reference to the recent block is known or agreed upon. ^21^ For instance, say the block hash of the recent block *B _N_* is known to be *δ _n_.* If data in block B1 is altered, its block hash is altered, eventually altering the block hash of *B _N_.* Hence, blockchain securely stores data to prevent alteration once recorded unless unanimous agreement exists among all parties involved. ^22^ In other words, the property of data immutability is achieved through a consensus mechanism that ensures all transactions are verified and added to the blockchain transparently and irreversibly. The result is blockchain provides a trustworthy ledger where data integrity and transaction history are preserved, fostering transparency and reliability in various applications such as supply chain management, financial transactions, and voting systems (i.e., the approval of above 50% of the network nodes is required). ^23^ De Filippi and Hassan ^23^ considers blockchain as a decentralized database (or state machine) that considers cryptographic primitives for the surety of data integrity and authenticity. It has also been known as electronic and decentralized, sometimes as immutable transaction ledger that provides cryptographic verification. These definitions underscore the fundamental aspects of blockchain technology, emphasizing its decentralized nature, cryptographic security measures, and the immutability of transaction records. ^20^

### 2.3 Blockchain Network

One of the important aspects of a blockchain ledger is what makes a valid data block and who can add a new one. A mechanism to add only a verified data block to the chain can aid in maintaining the perpetual integrity of the data. ^21^ This security feature is provided by sharing the blockchain among a special group of computing nodes known as a blockchain network. The broadcast network of computing nodes is known as a blockchain network (BCN). ^21^ It has capability to maintain a copy of a synchronized storage, which is defined as blockchain. Each node in a blockchain network runs a protocol for maintaining data consistency/integrity, verifying a transaction before it is added to the blockchain, maintaining operation transparency, and maintaining privacy through anonymity. A blockchain protocol permits anyone to propagate transactions through the network for verification and to be recorded in the ledger. Some special nodes, known as miners, verify and add well-formed transactions to the ledger. The miners in the network use consensus algorithms, such as Proof of Work (PoW), and Proof of State (PoS), to agree upon the block to be added to the blockchain. ^20, 24^ Since multiple verifiers verify the validity of a transaction, blockchain technology (blockchain and blockchain network) offers decentralization and disinter-mediation of transactions between users internationally. ^25^ In brief, blockchain technology provides a trustworthy ledger where data integrity and transaction history are preserved, fostering transparency and reliability in various applications such as financial transactions, supply chain management, and voting systems (i.e., the approval of above 50% of the network nodes is required). ^23^

**Figure 3.**
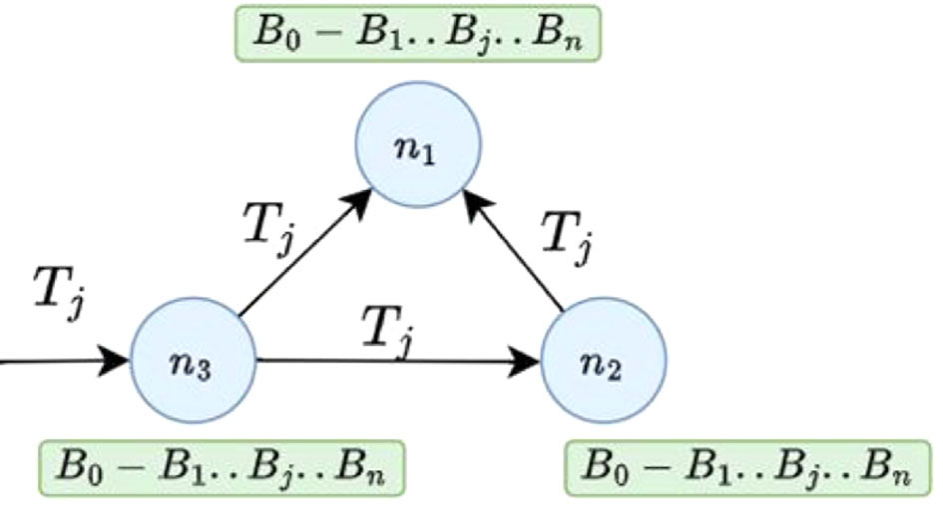
A simple network of blockchain network with three nodes. ^19^ This figure has been reproduced with permission from Adhikari et al. (2023). ^19^

Figure 3 a simple blockchain network with three network nodes *n*_1_, *n*_2_, and *n*_3_. Each node in a blockchain network maintains a copy of the underlying blockchain ledger *B*_0_ *← B*_1_ *← … ← B _j_.. ← B _n_.* If a transaction *T _j_* is sent to node n3 for validation and to be recorded in the existing blockchain, it is broadcast to connected nodes, which will also verify its correctness. ^19^ Many authors ^20, 24^ agree that blockchain allows anyone worldwide to send or participate in transactions, providing greater assurance that they are engaging with the rightful owner. This immutability is achieved through a consensus mechanism that ensures that all transactions are verified and added to the blockchain transparently and irreversibly.

### 2.4 Blockchain with Electoral Integrity from a Governance and Democracy Perspective

Blockchain technology has captured their attention as scholars and policymakers seek to bolster integrity in elections. Dominating the conversations was the impact of digital technology on institutional trust and its relation to electoral systems and the confidence voters placed in those systems. ^26^ The available literature reveals that implementing blockchain in voting systems has advantages and challenges.

Defenders of blockchain note that its features, particularly decentralization and the inability to alter information, can remarkably enhance the transparency of elections. Ohize et al. ^27^ stress that mobile voting systems based on blockchain technology have the potential to eliminate voter fraud and increase turnout by providing a secure and verifiable system for voting. They highlight that blockchain-based voting systems can be audited by anybody, enhancing credibility and faith in the system and electoral framework. Regardless of these advantages, Assouline et al. ^28^ argue that some privacy issues, cybersecurity, digital skills, and the exclusion of certain demographics add to the criticism of using blockchain technology in elections. There needs to be sufficient public awareness and development of the relevant technology to enable everyone’s participation.

Similarly, Farooq et al. ^29^ explain how blockchain can help protect voter registration information by making alterations transparent and traceable. Jafar et al. ^30^ discussed that electronic voting machines with blockchain technology could issue a printed paper receipt to the voters, which would aid in audits and boost trust in the vote-counting process. Moreover, Olaniyi et al. ^31^ underscore the risks of applying new technologies to the election process. They contend that elections are critical undertakings wherein things cannot go wrong since poorly applied blockchain systems would lose public trust. Implementing any technological application should be preceded by robust public awareness campaigns managed by reputable, non-partisan election officials.

The use of blockchain in the 2023 elections in Guatemala is an example that illustrates the expected results of the implementation. As stated in a Democracy and Society ^32^ report, the technology was applied to restore trust in the electoral system after allegations of fraud in preceding elections. Storing each transaction in a ledger using blockchain technology boosted the electoral system by easily detecting attempted manipulations.

One of the distinguishing features of democracy is transparency and digital democracy. As noted by many researchers, blockchain technology requires a guarded approach regarding security and public trust. ^33^ These two elements demonstrate the utmost necessity in maintaining the public’s trust in the system. Transparency of information coupled with accessibility indicates an open, robust form of government, which can be achieved through Internet technology. With innovative ideas purchasable through the internet, civic engagement engages us to welcome and engage in democracy through active voting. The exercise of voting becomes a civic duty for citizens of democratic countries as it incredibly directly sources their choices and decisions.

In this regard, elections are a striking example of implementing civic engagement. However, they tend to ignore that civic engagement can be defined in a multi-dimensional framework that features systems thinking that captures the complexity of social systems, making a participatory approach more appealing. Regardless of who utilizes the notion of digital democracy alongside open government, the digitally achieved or rather opened democracy acts as a form of power that the government can exercise and aid in refining the understanding of democracy through freedom.

***2.4.1 Theoretical Foundations and Potential Benefits***

A blockchain functions as a transparent public ledger incapable of modification, which opens the possibility for a new method of ensuring fair elections. Employing specific coding techniques, voting systems based on blockchain technology can offer End-to-End Verifiable (E2E) elections. ^34^ E2E implies that votes are cast on time, midstream, stored, and counted while stored. This capability can resolve problems associated with election fraud, risk of hacking, and personnel error. However, adding blockchain does not automatically provide more trust in elections. ^35^ Rather, the trust in blockchain voting is founded on the perception of voters, political leaders, and election officers. Without trust and knowledge of such systems, citizens cannot endorse democracy.

***2.4.2 Trust in Institutions and Electoral Legitimacy***

Trust in institutions is one of the central issues in political science. To what degree do people consider that political institutions are executing their roles properly? Standard voting systems dependent on institutions like election commissions, political parties, and independent monitors for free and fair elections rely on multiple functions working synergistically to acquire an equilibrium bloc. ^36^ It’s more a question of voters’ trust in the technology than in existing institutions and whether blockchain technology, which offers a mechanism without a need for a central authority, will make freer elections possible.

Most studies indicate a general decline in confidence in voting systems across the globe. People are apprehensive regarding cheating, vote tampering, and cybersecurity threats. ^37– 39^ Blockchain is suggested as an answer to this problem because there is no need to trust a central authority. All the transactions (votes) can be independently verified. Political analysts say elections are primarily social and not purely technical. ^40^ If people remain doubtful about blockchain security, ease of use, and the possibility of government tampering, increased reliability of elections will not be guaranteed.

Research indicates mixed results on users’ trust in voting via the Internet. Some suggest that younger, more adept technology users will trust elections run on blockchains while older users grapple with the system’s clarity and transparency. Moreover, in cases where there is extreme political division within parties, new election platforms are likely to be more divisive than unifying.

***2.4.3 Voter Perceptions and Digital Transparency***

Public trust in the fairness and accessibility of the electoral processes is fundamental to their credibility. A blockchain framework could worsen fears and misunderstandings without clarity, education, and transparency.

**Perceived Opacity vs. Actual Transparency**

Although blockchain can improve transparency by allowing voting processes to be publicly verified, most voters lack the technical skills necessary to audit blockchain systems. ^41^^,^^42^ Without straightforward verification mechanisms, blockchain implementation could inadvertently generate a black-box scenario where the community must depend on trusted specialists to navigate the opaque system instead of analyzing the results independently.

**Digital Literacy and Accessibility**

The positive impact of blockchain technology on electoral integrity relies on the assumption that all voters have the requisite digital literacy skills to engage with a blockchain voting system. Nonetheless, some studies indicate that the digital divides informed by education, age, and socioeconomic status can extend existing disenfranchisement. ^43^ That said, implementing blockchain voting needs to be preceded by efforts to educate voters about the technology so that they have adequate understanding and trust in the system.

**Role of Social Media and Disinformation**

The capability of blockchain technology to secure electoral data does not address the challenges of misinformation. Today’s elections are as much about the social media narratives, spin, and misinformation surrounding them as they are about casting votes. ^44^ Even if there were a voting system based on blockchain that was utterly impenetrable, trust in the system would still be undermined in highly polarised elections due to rampant inaccurate claims regarding the results.

***2.4.4 The Role of Digital Transparency in Democratic Legitimacy***

Democratic legitimacy defines whether a government has the right to rule and if it acts with the consent of the citizens. Improving digital transparency with blockchain technology may help in improving legitimacy by:

• Offering tangible proof that hacking did not occur with the voting.
• Minimizing dependence on authoritative sides reduces the chance of bias from the institutions.
• Increasing the possibility of international observation of elections, allowing outsiders to witness the processes live.

Elections conducted using blockchain technology may expose previously undetected discrepancies in the voting process. These discrepancies, however, could be misinterpreted as fraudulent activity, thus eroding trust in the electoral system. While blockchain may reinforce election integrity, its effectiveness will hinge on public perception, institutional support, and integration into current electoral systems. ^45^ Should insufficient public confidence and trust exist, an inclusive strategy could lead to opposition to the elections. Lastly, to bolster the democratic image of a nation, issues surrounding democratic election integrity need comprehensive public trust initiatives and civic education campaigns that examine governance issues, establish trust in the system, and systematically approach the integration of blockchain technology.

## 3. Related work

The first electronic voting system was conceptualized by “David Shaum” in the early eighties, where public-key cryptography was utilized to ensure that electors were selected anonymously and to separate the voters’ identity from the ballots. ^46^ Estonia has adopted the electronic voting system for its national election in 2005. ^47, 48^ However, Denmark’s Liberal Alliance in 2014, started planning to integrate the blockchain to enhance the privacy and security of national elections in Denmark. ^7^

During the later months of 2018, South Korea’s National Election Commission introduced a trial of a blockchain-based online voting system for the South Korean elections, aiming to enhance the reliability and security of the voting system. As a cutting-edge innovation rooted in robust cryptographic principles, blockchain technology facilitates easier data access for candidates and observers, contributing to greater transparency and trust in the electoral process. However, they believe that applying blockchain-based voting will raise the rate of buying votes. As explained in the previous section, blockchain technology can validate whether a bribed voter adhered to their part of the deal or not. ^49^ Overall, the trial of using blockchain-based technology in electronic voting has been successful.

This foundation empowers blockchain-based applications to capitalize on advanced cryptographic techniques, enabling them to implement highly adaptable security solutions. By leveraging these cryptographic abilities, blockchain enhances the security of data and transactions and ensures transparency and integrity across decentralized networks. ^1^ Nearly all discussions about blockchain converge on a shared concept, though articulated with variations.

Experts believe the traditional voting system has problems like the security of the votes; hence, blockchain is the perfect solution for securing elections. Hence, technologist consider it an ideal platform for global democratic systems. ^12^ Blockchain technology in the electronic voting system protects the secrecy of the ballots and is ubiquitous. Further, the free and open source peer review software allow a free and independent audit of the result and decreases the confidence level required from the organization or the election entity. ^50^

The distributed ledger technology allows multiple points to have the ledger instead of restricting to one location; this means that all points can have the same results, unlike in traditional ballot boxes. ^12^ Therefore, failure is rare because it is decentralized, and there is a rare chance of being corrupted. Different transactions have blocks holding previous electors’ transactions to ensure the voting validity, security, and connectivity. If any of the blocks are compromised due to a lack of connection between the blocks, it will be easy to detect. Real voting takes place in blockchain. The votes by voters get sent to one of blockchain’s nodes in the voting system, which then adds the vote to the blockchain. The voting system has a node in each area to ensure the decentralization of the democratic framework. ^7^ Blockchain technology safeguards election records from illegal access, altering, and changing of ballots. ^1^ The consensus algorithm makes the blockchain dominant over other technologies, and hence, it has increased the adaptability of the blockchain as it can operate tasks faster, more accurately, and more efficiently than bureaucratic systems based on paper voting. ^12, 51^

As technology advanced, many countries began integrating blockchain technology into their voting systems to enhance transparency, privacy, and accessibility in the voting process. ^52, 53^ Furthermore, many countries adopt blockchain technology in their e-government systems. ^54^ However, the integrity of the voting process hinges on a consortium of trusted validators tasked with either approving or rejecting transactions. A recent study by Ref. 55 proposed an innovative adjustable blockchain that can enhance the voting procedure and mechanism, and address security concerns. Their proposed framework offers an enhanced and more secure electronic voting process, ensuring data integrity through advanced hashing technology. Blockchain technology facilitates easier data access for candidates and observers, contributing to greater transparency and trust in the electoral process. ^49^

Blockchain technology is increasingly recognized as a cutting-edge innovation rooted in robust cryptographic principles. This foundation empowers blockchain-based applications to capitalize on advanced cryptographic techniques, enabling them to implement highly adaptable security solutions. By leveraging these cryptographic abilities, blockchain enhances the security of data and transactions and ensures transparency and integrity across decentralized networks. ^56^ Nearly all discussions about blockchain converge on a shared concept, though articulated with variations. According to the paper by De Filippi and Hassan, ^23^ it is described as a “decentralized database (or state machine) that relies on a set of cryptographic primitives to ensure data integrity and authenticity.” Blockchain is further defined as an electronic, decentralized, immutable transaction ledger that provides cryptographic verification. These definitions underscore the fundamental aspects of blockchain technology, emphasizing its decentralized nature, cryptographic security measures, and the immutability of transaction records. ^20^

Many authors agree that ^20, 24^ blockchain allows anyone in the world to send or participate in transactions, providing greater assurance that they are engaging with the rightful owner. The blockchain securely stores data to prevent alteration once recorded unless unanimous agreement exists among all parties involved. ^22^ This immutability is achieved through a consensus mechanism that ensures all transactions are verified and added to the blockchain transparently and irreversibly. As a result, blockchain provides a trustworthy ledger where data integrity and transaction history are preserved, fostering transparency and reliability in various applications such as financial transactions, supply chain management, and voting systems. ^23^ Blockchain technology offers decentralization and disinter-mediation of transactions between users internationally. ^25^ For a voting system utilizing blockchain, real voting takes place in a blockchain network. The votes by voters get sent to one of blockchain’s nodes on the voting system, which then adds the vote to the blockchain. The voting system has a node in each area to ensure the decentralization of the democratic framework. ^7^ Blockchain technology safeguards election records from illegal access, altering, and changing of ballots. ^1^ This technology remains prominent due to the consensus algorithm that makes it difficult to vote transactions and makes it difficult to alter or forge. ^51^

Using immutable storage, you can securely manage and maintain outsourced computational ledgers, protecting them against tampering and unauthorized access. ^57^ The work integrates blockchain and machine learning for secure data transmission, employs NuCypher proxy re-encryption for efficient neighborhood encryption without cipher conversion, and utilizes ANN to optimize data delivery and record management in smart cities. ^58^A blockchain framework enabled by Hyperledger Sawtooth, offering a secure and trusted execution environment where service delivery mechanisms and protocols ensure immutable ledger storage security, along with peer-to-peer network communication for both on-chain and off-chain industrial activities. ^59^ This study proposes a lightweight Plenum consensus algorithm (BLPCA) for consortium blockchains on Hyperledger Indy, leveraging optimized Byzantine Fault Tolerance to manage large-scale decentralized socioeconomic tax traffic within hierarchical systems efficiently. ^60^ The study highlights the benefits of a permissioned private blockchain, addresses the challenges of the Proof-of- Elapsed Time (PoET) consensus mechanism in distributed applications, and proposes a lightweight middleware consensus called “B-LPoET” to improve blockchain adaptability for private chains. ^61^ In various domains blockchain plays a critical role; hence it has potential in the voting system. However, there are limitation in the traditional voting systems that needs to be addressed by the secure voting system based on blockchain.

## 4. Methods

This research adopts a quantitative approach and employs an experimental design methodology. It operates the voting systems with synthetic population data using open-source software. The dataset includes citizens’ National IDs and basic demographic information and is publicly accessible through the provided URL [1].

Oman’s electronic voting system is structured around diverse system models and innovative methods to enhance the efficiency, transparency, and accessibility of electoral processes nationwide. These models encompass centralized, decentralized, and hybrid approaches, each tailored to address specific requirements and challenges within Oman’s electoral framework. ^62^ In the centralized model, a single authority oversees all aspects of the electronic voting process, ensuring uniformity and centralized control over voter registration, ballot distribution, and result aggregation. ^63^ The proposed model emphasizes centralized security measures and operational oversight to safeguard against potential threats and ensure the integrity of the electoral outcome. Conversely, decentralized systems distribute authority and processing power across multiple nodes or centers within Oman. This approach enhances resilience against localized disruptions or attacks, as voting operations can continue independently across different regions. ^64^ Decentralized models, such as blockchain, often require robust coordination mechanisms to synchronize voter data and ensure consistent standards of security and accuracy throughout the electoral process. Addressing these challenges is crucial for improving elections’ efficiency, security, and inclusivity, with blockchain technology offering potential solutions to many of these issues. ^65^ Hybrid systems in Oman blend elements of both centralized and decentralized models, offering flexibility and redundancy while maintaining centralized oversight over critical electoral functions. This model allows for adaptive responses to varying operational conditions and enhances the overall reliability and resilience of the voting system. Key methods employed in Oman’s electronic voting system include advanced biometric authentication technologies to verify voter identities securely. Biometric methods such as fingerprint scanning or facial recognition help mitigate fraud risks and ensure that each vote is cast by the rightful individual, enhancing overall confidence in the electoral process.

The proposed model based on blockchain technology plays a pivotal role in Oman’s electronic voting system by providing a secure and transparent method of recording and verifying votes. The system helps to ensure their security and reliability as existing e-voting systems lack a clear protocol, making it difficult to ensure their security and reliability. The system utilizes the blockchain’s distributed ledger to securely record and verify each vote. Unlike traditional centralized voting systems where trust is placed in a single authority, blockchain-based voting ensures that all transactions (such as voter registration, validation, voting, and counting) are transparently recorded and cannot be altered retroactively. Each vote is cryptographically secured, linked to previous transactions, and distributed across a network of computers (nodes), making it extremely difficult for malicious actors to manipulate or manipulate voting data. Blockchain’s decentralized ledger ensures the immutability and transparency of voting records, making them highly resistant to tampering or manipulation and eliminates the need for intermediaries and central authorities, fostering trust among participants and reducing the risks of fraud or coercion. This aspect is particularly valuable in participatory voting systems, where ensuring the integrity of each vote is paramount to maintaining the democratic process’s legitimacy. The decentralized storage of voting data ensures the integrity and confidentiality of voting data, and Oman’s electronic voting system employs robust encryption techniques and stringent security protocols. These measures protect sensitive information from unauthorized access or manipulation, maintaining the privacy and trustworthiness of the electoral process. In addition, audit mechanisms allow for the verification and scrutiny of all actions taken during the voting process, ensuring adherence to established protocols and regulations.

Ethereum offers a highly secure and transparent foundation for electoral processes, ensuring trust in digital voting systems. Its smart contract automation eliminates the need for intermediaries, reducing human errors and minimizing the risk of election fraud. ^30^ By leveraging a decentralized network, Ethereum ensures that no single entity controls the voting process, making tampering or manipulation virtually impossible. Additionally, the immutability of its blockchain enhances public confidence by recording every vote on a transparent ledger that cannot be altered.

Ethereum has limitations regarding transaction scaling, a factor critical to the effective, secure, and economic infrastructure underpinning voting systems. ^66^ An estimated 15-30 transactions per second (TPS) is what Ethereum can achieve succinctly in its current settings, which is abysmally low for elections that have millions of voters. This too unrestricted scenario would cause a complete and catastrophic collapse for the network, higher fees and processing delays would cause significant issues, and slower vote acceptance mechanisms would result in security risks. ^67^ Ethereum’s network has faced catastrophic congestion spikes in high-demand periods. Such an election requires consolidation of Ethereum’s voting systems, which require instantaneous and secure logging of millions of votes. Crippling slowdowns under such intense pressure could result in a complete loss of voter faith, raising alarm bells about the legitimacy of the automotive voting process. Without vast alterations, the inefficient management of explosive transaction volume would render change publicized elections without overhaul infeasible.

Furthermore, Ethereum’s ongoing scalability improvements, particularly through Layer 2 solutions such as Rollups and Polygon, significantly enhance its transaction speed and cost efficiency, making it more viable for large-scale elections. ^67^ Research and developers have been focusing on Layer 2 scaling solutions and sharding, which seek to optimize transaction load distribution, as potential approaches to alleviating Ethereum’s scalability issues. Additional protocols that increase transaction throughput and are based on the main Ethereum blockchain are called Layer 2 protocols (L2). The optimistic rollups assume all submitted transactions are valid without preliminary checks and only run computational checks if there is a dispute. They reduce expenses like ZK Rollups perform off-chain transaction verifications before final on-chain submissions to ensure that only legitimate submissions are included. ^68^ Rollups involve combining several transactions into a single one, and this combined transaction is what is submitted to the main blockchain. A subset of voters can initiate and process transactions off-chain; only the final result is committed to Ethereum. This provides considerable relief to the network. Blockchains other than Ethereum that run in parallel to Ethereum are called sidechains. They can maintain their ledgers and, in conjunction with, periodically merge with the primary Ethereum blockchain. This sidesteps congestion.

Parallel processing through shards is yet another potential solution. It divides the Ethereum blockchain into smaller constituent chains (shards) that can individually execute transactions and manage smart contracts. It permits parallel execution and increases scalability while ensuring that voter transactions are processed seamlessly concerning network congestion. ^69^

The system’s use case model further explains its concepts and key actors that make it successful. ^12^^,^^50^

### 4.1 Use case model

**Figure 4.**
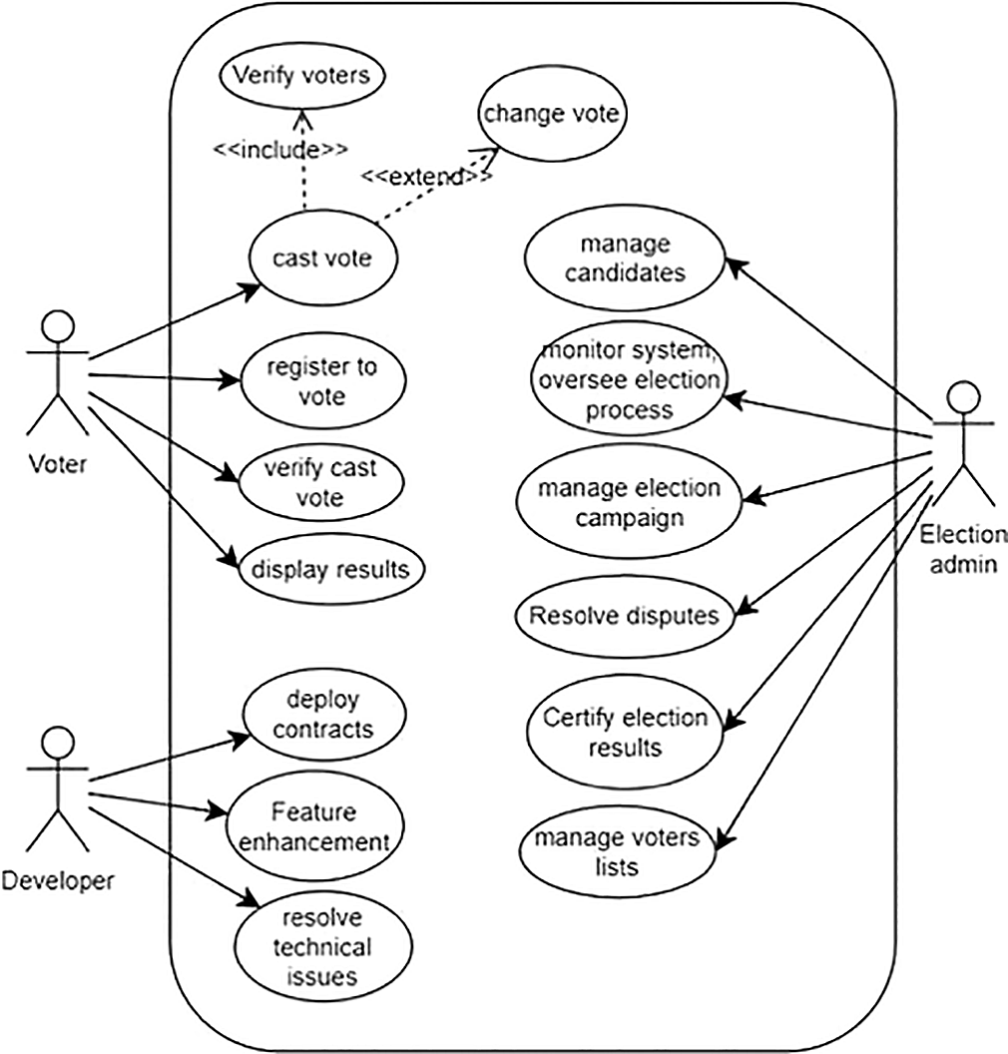
Use case model of the system.

The proposed blockchain-based voting system involves key actors: election administrators, voters, election auditors, Ethereum nodes, and smart contract developers. Election administrators manage the process, voters cast secure electronic votes, auditors ensure integrity, Ethereum nodes maintain the network, and developers create smart contracts. Figure 4 illustrates a simplified use-case model of the system, depicting interactions among these actors and the blockchain infrastructure, enhancing transparency and security in electoral processes.

**Election Administrators** The main operations of election administrators are as follows:

• **Manage Election:** Administrators can create, configure, and manage election campaigns. This includes setting up the parameters such as voting dates, eligible candidates, and voter eligibility criteria.
• **Monitor System:** Administrators can monitor the overall system health, including blockchain network status, transaction verification, costs, and any potential security threats.
• **Generate Reports:** Administrators may need to generate various reports on election results, voter turnout, and system performance for auditing and analysis.
• **Oversee Election Process:** Election authorities can oversee the entire election process, ensuring fairness, transparency, and compliance with electoral regulations.
• **Resolve Disputes:** Election authorities can intervene and resolve disputes or challenges related to voter eligibility, ballot integrity, or other election-related issues.
• **Certify Election Results:** Election authorities can certify the final election results based on the data recorded on the blockchain ledger, providing official validation of the outcome.

**Voters** Voters are the general public illegible for a voting campaign. The main operations of voters are as follows:

• **Register to Vote:** Voters can register themselves on the blockchain-based system to participate in elections. This process may involve identity verification and confirmation of eligibility criteria. To simplify the process, voters can register to the blockchain system for a voting campaign via an intermediate web application server operated by a government body such as the election commission.
• **Cast Vote:** Voters can securely cast their votes for their chosen candidates. The system should ensure anonymity, integrity, and verification of each vote.
• **Verify Vote:** Voters can verify that their vote has been correctly recorded on the blockchain ledger, providing transparency and confidence in the voting process.
• **View Candidate’s details:** Voters can access information about candidates, their agendas, and other relevant details to make informed voting decisions.

**Auditors**

• **Audit System Integrity:** Auditors can verify the integrity of the smart contracts and voter registration, ensuring that the system operates without any manipulation or fraudulent activities.
• **Review Transaction History:** Auditors can review the transaction history stored on the blockchain ledger to validate the accuracy and transparency of the voting process.
• **Verify Compliance:** Auditors can ensure the system complies with relevant regulations and standards governing elections, data privacy, and security.

**Web Application and Smart-Contract Developers**

• **Develop Smart Contracts:** Developers can create and deploy smart contracts that govern the rules and logic of the voting system, ensuring transparency, security, and automation of voting processes.
• **Enhance System Features:** Developers can propose and implement enhancements to the blockchain-based voting system to improve scalability, usability, and security.
• **Resolve Technical Issues:** Developers can troubleshoot and resolve technical issues within the system, such as bugs, performance bottlenecks, or vulnerabilities.

**Ethereum Nodes** Ethereum nodes play a prominent role in the blockchain-based voting system by actively listening for and processing critical transactions such as voter verification, vote casting, and election result dissemination. Their active participation is integral to maintaining the integrity and transparency of the electoral process. Therefore, Ethereum nodes are considered principal actors within our system, ensuring secure and decentralized management of voting operations. This collaborative effort among nodes strengthens the reliability and resilience of the voting system, bolstering confidence in the accuracy and fairness of election outcomes.

• **Verify voters:** In Ethereum-based voting systems, nodes verify voter identities before they can cast their votes. Nodes maintain the blockchain’s integrity by executing smart contracts that authenticate voters and record their votes securely. This decentralized approach ensures transparency and safeguards against manipulation, promoting trust in the electoral process.
• **Receive cast votes:** In Ethereum-based voting systems, Ethereum nodes receive and validate votes cast by voters for candidates, ensuring the integrity and transparency of the voting process through decentralized verification and blockchain recording.
• **Display/Print results:** In Ethereum-based voting systems, Ethereum nodes validate votes, tally election results, and securely record the outcome on the blockchain, ensuring transparency and immutability in the electoral process.

**System Architecture** The architecture of a simplified blockchain-based distributed voting system comprises four tiers: the storage services tier (Tier-1) ensures secure storage of encrypted voter data and transaction records, collectively enhancing transparency, security, and reliability in electoral operations. The Web3 Blockchain network smart contract services tier (Tier-2) hosts smart contracts governing the voting process. The Web2 application services tier (Tier-3) manages business logic and user authentication. The front-end tier (Tier-4) provides user interfaces for voter interactions.

**Tier-1**

**Figure 5.**
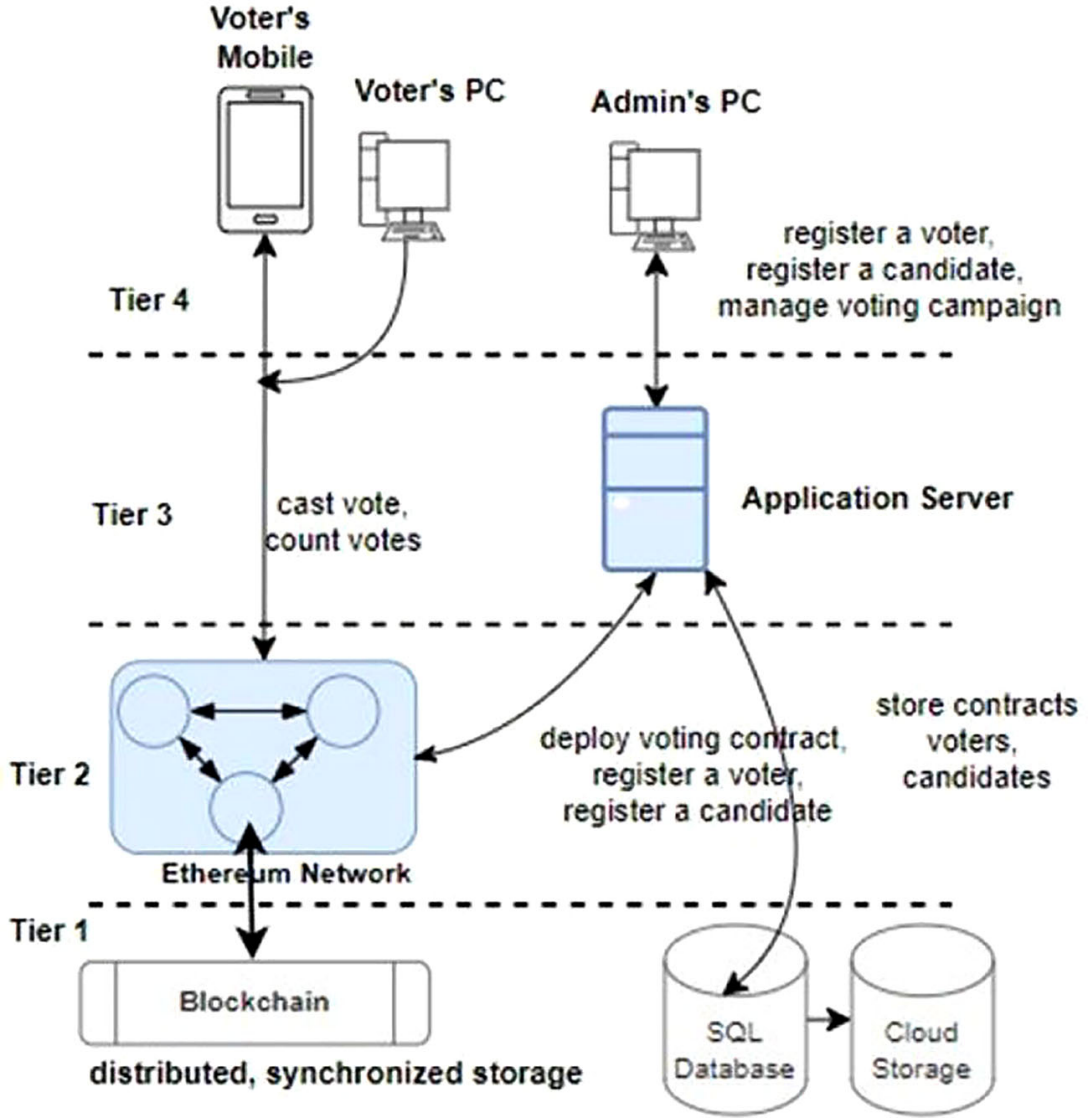
A simplified Blockchain-based distributed voting system architecture.

As illustrated in Figure 5, tier-1 encompasses critical data storage services like MySQL for traditional data management and blockchain ledger for immutable, decentralized record-keeping. Tier-3 voting application server utilizes MySQL-based storage engines to efficiently store and retrieve user credentials, voter information, and administrative data. Meanwhile, the Ethereum network in tier-2 involves Ethereum nodes maintaining individual copies of the blockchain ledger that guarantees transparency, security, and integrity in the voting process by securely recording each vote as a tamper-proof transaction. This Tier-1 layer forms the foundation of the application’s architecture, combining the reliability of traditional databases with the innovation and trust of blockchain technology to uphold the integrity of the voting process. In other words, a blockchain ledger ensures that a voter can only cast a single vote for a candidate by restricting the backtracking of the cast votes to the candidates and producing trustworthy voting campaign results.

**Tier-2**

Tier 2 is the critical service layer of a trust-less distributed voting system. It is composed of an Ethereum blockchain network. An Ethereum network consists of thousands of computing nodes that individually store a copy of a distributed, synchronized database known as blockchain [citation]. The network is a distributed, peer-to-peer network. ^70^ The major feature of such a network is that each Ethereum node can store smart contracts in the blockchain and run them in its Ethereum virtual machine. A smart contract is a stored procedure that performs atomic digital transactions, the outcome of which can be verified by the participating Ethereum nodes and committed in the blockchain database. Every transaction is stored in the blockchain network such as Ethereum, and such storage is immutable and temper-proof. ^71^

The Ethereum network provides the following services to our voting system:

1. Register voters and store them in the blockchain.
2. Register and store election officers and election administrator mandates in the blockchain.
3. Register a valid vote cast by a registered voter for a candidate
4. Respond to the user’s query to check if a voter is registered.
5. Respond to the user’s query to check the count of votes received by a candidate.

The operations performed all in the smart contract deployed in the Ethereum network.

**Tier-3**

Tier 3 is composed of the web application services that can be utilized by election administrators for the following tasks:

1. Register the voting campaign officers.
2. Register the voters for the voting.
3. Register the candidates for the voting campaign.
4. Deploy the voting campaign smart contracts to the Ethereum blockchain.

Tier-3 application servers utilize Tier-1 relational database services such as MySQL for data storage. Our tier-3 web application service was built in C#. Alternatively, we can use cloud storage services such as database.com, Oracle, Amazon Aurora, IBM Db2, and BigQuery. ^72^

**Tier-4**

Tier 4 architecture encompasses various end-user devices, including smartphones, personal computers, tablets, and client applications such as web browsers and crypto-wallets. These devices play a pivotal role in the blockchain-based voting system by serving as interfaces through which stakeholders, including voters and election administrators, interact with the electoral process. One of the distinctive features of devices in this layer is their ability to securely host and manage crypto wallets. Crypto wallets store cryptographic keys that authenticate users and authorize their actions within the voting system. For voters, these wallets enable secure and anonymous casting of votes, ensuring confidentiality and integrity throughout the voting process.

Election administrators utilize crypto wallets to manage voter registration, monitor election activities, and validate results, maintaining transparency and accountability in electoral operations. Moreover, end-user devices in tier 4 facilitate seamless connectivity to the Web3 blockchain network, where smart contracts execute and enforce the voting process rules. This direct interaction with the blockchain ensures that all transactions, from voter registrations to ballot submissions and result tabulations, are recorded immutably and transparently on the distributed ledger. By leveraging the capabilities of smartphones, computers, and other client applications, the voting system enhances accessibility for voters, enabling them to participate conveniently from any location with internet access. This inclusivity fosters higher voter turnout and engagement in democratic processes, while the secure handling of crypto wallets and blockchain interactions ensures the system’s resilience against cyber threats and fraud. The voters utilize the client applications for the following tasks:

1. To cast the votes for the candidates.
2. To register for the voting campaign.

We created programs based on .Net Core 7 to simulate the operations of the tier-4 devices and applications. However, browser-based client applications can be developed for production purposes to support multiple operating systems, such as Windows, MacOS, Linux, Android and iOS.

## 5. Experimentation setup

Researchers developed a simulation package in the *C*# .Net platform to determine the proposed system’s efficiency. The smart contract was developed in *C*# and deployed in the Ethereum-based Geth test chain provided by Nethereum. The blockchain network Geth is deployed on a machine with Microsoft Windows 10 Enterprise OS, a 12th Gen Intel ^®^ Core™ Intel i7-12700, 2100 Mhz, 12 Cores processor, and 16GB installed Physical Memory (RAM).

For experimentation, a testbed based was established using the Geth test chain and .Net Core clients, integrated with Ethereum through Nethereum a .Net library designed for blockchain connectivity [2]. The library contains APIs that simplify the access and smart contract integration with Ethereum nodes. In the context of communication design, the protocol is HTTP, which can be switched to HTTPS for a secure channel. However, to simplify communication, the protocol was designed to be HTTP-based. The algorithms described in this section were implemented using *C*#.Net.

Algorithm 1. **SmartContract *π.***

1: **Struct:** Election {uint eid; uint year; string name; uint type}

2: **Storage:**

3:   address ContractOwner

4:   Elections *←* MAP (address => Election);

5:   Voters *←* MAP (address => uint);

6:   Candidates *←* MAP (address => uint);

7:   Voters *←* MAP (address => uint);

8: **procedure** CandidateRegistration (address ID)

9: **if** Candidates [ID] **then**

10:   return FALSE

11: **end if**

12: **if** ContractOwner == Message. Sender **then**

13:   return FALSE

14: **end if**

15:   Candidates [ID] = TRUE

16: **end-procedure
**

17: **procedure** VotersRegistration (address ID)

18: **if** Voters [ID] **then**

19:   return FALSE

20: **end if**

21: **if** ContractOwner == Message. Sender **then**

22:   return FALSE

23: **end if**

24:   Voters [ID] = TRUE

25: **end-procedure
**

26: **procedure** CastVote (address ID)

27: **if** Voters [ID] == TRUE **then**

28:   return FALSE/*voter already voted*/

29: **end if**

30: **if** Candidates [ID] **then**

31:   return FALSE/*candidate is registered.*/

32: **end if**

33:   Candidates [ID] += 1

34:   return TRUE

35: **end-procedure
**

36: **procedure** VoteCount (address ID)

37: **if** Candidates [ID] **then**

38:   return Candidates [ID]

39: **end if**

40:   return FALSE

41: **end-procedure
**

42: **procedure** Constructor()

43:   ContactOwner = Message.Sender

44:   return TRUE

45: **end-procedure
**

Algorithm 2. **DeploySmartContract.**

1: **Inputs:** EthereumNetwork *chi ←* ( *networkid* = *{}, url* = *{}*), SmartContract *π*/*Hereafter, (..) is a tuple defining the object*/

     /*{..} is a place-holder for appropriate values.*/

2: **Outputs:** SContractReceipt *r ← (time, contract-address, message)*

3: *v.*connect_to( *χ*); /*connect the the blockchain network network χ*/

4: *r ← χ.*deploy( *π*); /*execute a transaction to deploy the smart contract *π* on the blockchain network χ*/

Algorithm 3. **CandidateRegistration.**

1: **Inputs:** EthereumNetwork *χ*, SmartContract *π*

2: **Outputs:** CRegistrationReceipt *r ← (time, address, message)*

3: **Initialize candidates** *C ←* { *c*_0_, *c*_1_, .. *c _n_*}; *c _i_ ← (ID, name, SSN, dob, election-id)*

4: **for** *c* in *C* **do**:

5: *c.*connect_to( *χ, π*);

6: *r ← c.*cregister(); /*execute a transaction to register a candidate for an election with ID, eid.*/

7: **end for**

Algorithm 4. **VotersRegistration.**

1: **Inputs:** EthereumNetwork *χ*, SmartContract *π*

2: **Outputs:** RegistrationReceipt *r ← (time, address, message)*

3: **Initialize voters** *V ←* { *v*_0_, *v*_1_, .. *v _n_*}; *v _i_ ← (ID, name, SSN, dob, election-id)*

4: **for** *v* in *V* **do**:

5: *v.*connect_to( *χ, π*); /*establish the connection*/

                             /* to the network and the smart contract*/

6: *r ← v.*register(); /*execute a transaction to register a voter for an election with ID, eid.*/

7: **end for**

Algorithm 5. **CastVote.**

1: **Inputs:** EthereumNetwork *χ*, SmartContract *π*, Voters *V*, Candidates *C*

2: **Outputs:** VoteCastReceipt *r ← (time, address, message)*

3: **for** *v* in *V* **do**:

4: *v.*connect_to( *χ, π*);

5: *r ← v.*cast_voteto( *c*: *c ∈ C*); /*voter *v* voting the candidate *c ^′^* */

6: **end for**

## 6. Results and Discussion

Performance evaluation in a blockchain-based voting system involves assessing its efficiency, reliability, and scalability, focusing on metrics such as transaction throughput, latency, scalability under increasing loads, security, fault tolerance, resource utilization, audit ability, transparency, and user experience to ensure optimal system operation and voter trust.

As part of the prototyping, the following tasks were completed:

1. Load a 1000 list of voter-related data to SQL servers. Each voter is identified by a synthetic Social Security Number (SSN) and has other information such as full name, address, phone number, and email address.
2. A client program developed in *C*# .Net produced 1000 voting (casting votes) transactions encompassing 1000 total voters identified with synthetic Social Security Numbers (SSN) executed against the network.

**Figure 6.**
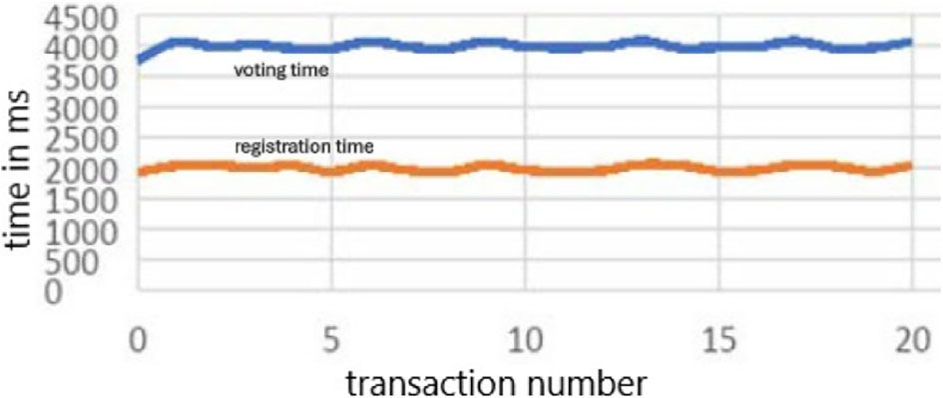
Transactions Times in Millisecond for registration and voting transaction.

Two main transactions, voter registration and vote casting, were executed in two different ways:

1. Synchronous transactions: This method synchronously submits transactions to the blockchain network. In other words, individual voter registration and individual vote-casting transactions were executed one after another. A total of 1000 transactions for each were executed to measure the execution time. Figure 6 shows the time required for registration and voting 20 individual transactions run synchronously.
2. Asynchronous transactions: This method submits transactions to the blockchain network asynchronously. The voter registration and vote-casting transactions were executed asynchronously in batch sizes of 10, 20, 30, and 50. For example, with a batch size of 10, 10 voter registration transactions and 10 vote-casting transactions were asynchronous. The final vote registration and vote casting time for each batch were measured. Figure 6 shows the time required to execute 1000 transactions for registration and voting. The red plot is the line plot for the time (MS) taken by transaction for voter registration, the grey line plot is for the time (in MS) taken by transaction for voting and receipt of the confirmation, and the blue line plot is the total time taken for both transactions. The spikes in time measurements in asynchronous transactions are often a result of the dynamic and distributed nature of asynchronous systems, where various factors such as network latency, resource contention, flow control queuing delays, and resource allocation and scheduling can influence transaction processing time.

**Figure 7.**
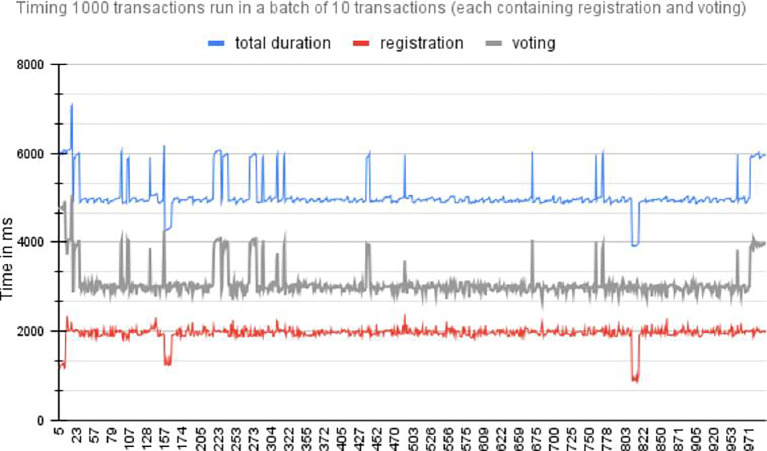
Performance per 1000 Transactions.

Figure 7 shows the time required by asynchronous execution of 100 batches, each consisting of 10 transactions for voter registration and vote casting. The red plot is the line plot for time (MS) taken by transaction for vote casting and receipt of the confirmation, and the blue line plot is the total time taken for voter registration.

**Figure 8.**
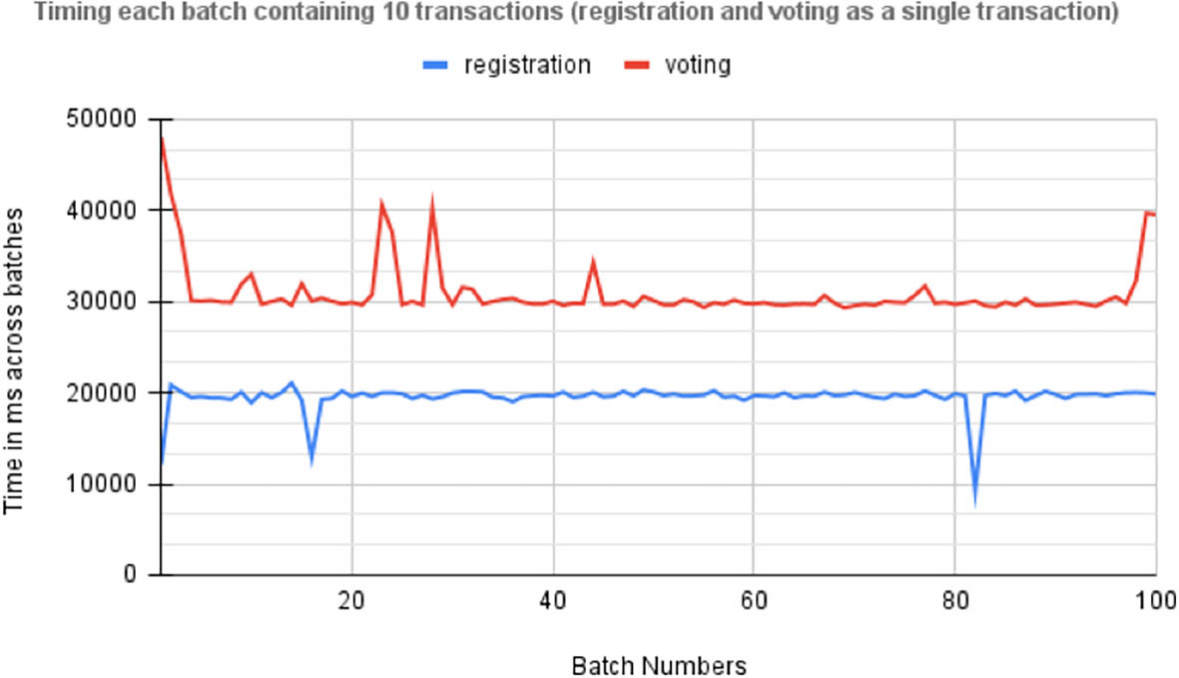
Times across batches each containing 100 transactions.

Figure 8 Timing 100 batches of transactions, each containing 10 individual transactions for registration and voting.

**Figure 9.**
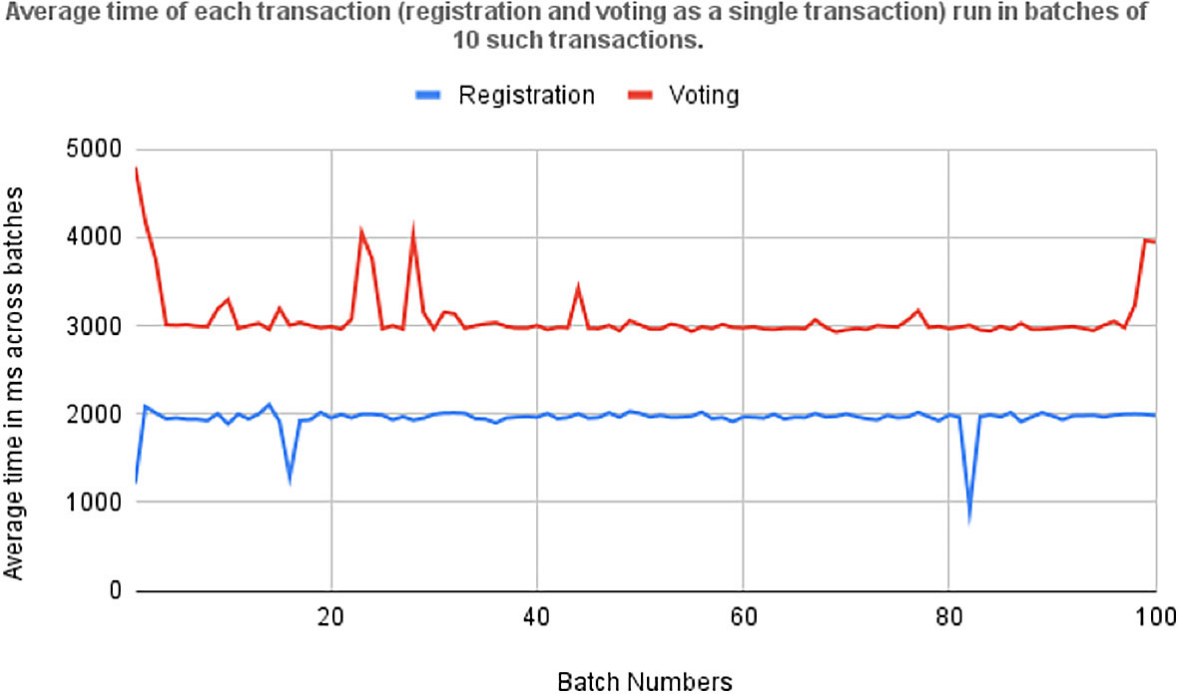
Average time required by asynchronous execution of 100 batches.

Figure 9 displays the average time required for the asynchronous execution of 100 batches, each containing 10 transactions for voter registration and vote casting. This metric is crucial for evaluating the efficiency of the blockchain-based voting system, particularly in handling concurrent transactions during electoral peaks. It underscores the responsiveness and reliability of end-user devices like smartphones and computers securely processing these transactions, highlighting the system’s scalability and performance under varying loads. These insights are essential for optimizing the voting system’s infrastructure to ensure robustness and effectiveness in electoral operations.

The experiment primarily focuses on the viability and processing time of election-related transactions. Security-related issues include security vulnerabilities, potential attack vectors, or the risk of blockchain manipulation. While blockchain is widely considered a secure and tamper-resistant technology, it is susceptible to several potential vulnerabilities and attack vectors such as Sybil attacks, 51% attacks, Smart Contract Vulnerabilities, data integrity and oracle manipulation, Denial of Service (DoS) attacks, Key Management and Insider Threats, Blockchain forking and inconsistency, ^73^ among others. ^74^ The most pertinent threats are discussed below:

• Sybil Attacks: In a Sybil attack, an adversary creates multiple fake identities to gain disproportionate influence over the network. This can compromise consensus decisions, particularly in permissionless blockchains. It can be mitigated by enforcing large stakes for validators in a network like Ethereum. ^75^
• 51% Attacks: A 51% attack occurs when a single entity or coalition controls most of the network’s computational or stake power, allowing them to manipulate the ledger (e.g., double-spending). While this risk is more common in public proof-of-work systems, we can minimize it using a practical Byzantine Fault Tolerant (PBFT) consensus algorithm among pre-approved validators, making it resilient against such attacks. ^73,^ ^76^
• Smart Contract Vulnerabilities: If smart contracts are used in the framework, they can introduce exploitable bugs (e.g., reentrancy, overflow/underflow errors, or improper access control). We can address this by enforcing rigorous contract auditing and adopting standardized development practices, such as those recommended by OpenZeppelin, and formal verification tools when feasible. ^77^
• Denial of Service (DoS) Attacks: DoS attacks can target validator nodes or clog the network with excessive transactions. To address this, we can implement rate-limiting, transaction prioritization, and resource-monitoring techniques, especially at the application layer. ^78^

## 7. Conclusion

It has been concluded that blockchain technology has enhanced the integrity and security of digital voting systems in Oman. Blockchain can significantly bolster voter trust and participation in the electoral process by addressing critical issues such as data integrity, fraud prevention, and transparency. While the advantages of such technology are widely discussed, there is scant evidence detailing public perception, especially about Omani citizens. It is important to know how Omanis view a blockchain-based voting system because public trust is critical to the success of any election reform. Studies conducted in Oman are lacking, and future work should focus on designing effective questionnaires and trust measurement scales that accurately determine voters’ trust in the technology. Estonia, where its e-governance systems, including its i-Voting system, were bolstered into blockchain technology. The adoption of blockchain has improved security and transparency, increasing public trust in digital voting. The Estonia example demonstrates the necessity of solid digital frameworks alongside a digitally connected and trusting populace. The blockchain-based voting framework ensures a tamper-proof and transparent voting system, maintains voter privacy and facilitates real-time auditing. Successful implementation, however, requires overcoming several challenges, including technological readiness, scalability, and societal resistance to change. The study highlights the necessity for a robust regulatory framework and comprehensive public awareness initiatives to foster acceptance and understanding of blockchain technology in the electoral context. Policymakers are urged to initiate pilot projects to evaluate the practical implications of blockchain integration, ensuring a gradual and informed transition. Ultimately, adapting blockchain in Oman’s electoral system could serve as a model for other nations seeking to enhance the security and credibility of their voting processes. Oman has the opportunity to set a precedent in electoral innovation, ensuring that its democratic processes are robust, transparent, and trustworthy. While the proposed model offers numerous benefits, there are risks and limitations to consider, including the need for advanced infrastructure and technical expertise, potential scalability issues as voter numbers grow, and resistance from the public and stakeholders unfamiliar with or concerned about the reliability of blockchain technology. Oman must analyze and draw lessons from other global practices to advance to a leading position in blockchain voting innovation. This goes beyond mobilizing the technology and logistics to examine the socio-political landscape of public trust. By conducting basic perception research on citizens, Oman can customize models compared to existing ones to fit its culture and politics. Such an approach would ensure that all technological changes important in elections would work and be endorsed by the people. The proposed model also has limitations that need to be considered [3]. For instance, the issue of scalability needs to be addressed as the number of voters increases substantially. Theoretically, the Ethereum Network supports 119.1 transactions per second (tx/s), which is relatively lower than other blockchain networks, such as Solana (65000 tx/s), Tron (2516 tx/s), Stellar (1137 tx/s), BNB Chain (2222 tx/s), and Polygon (714.1 tx/s). There are various scaling solutions, including sharding, off-chain computation, rollups (such as Optimistic or ZK-Rollups), and hardware acceleration, among others. Future work includes testing scalability and performance across various blockchain networks.

## Ethics and consent

This study does not involve human participants or animals, and therefore, ethics approval was not required. No sensitive data were used, and no real personal information was collected.

## Author contributions statement

The authors hereby confirm that we all have made a substantial contribution. A.K.S. has contributed to idea generation and provided direction for the research, and designed the methodology, and wrote the original draft. However, N. A, A.S.S are engaged in the experiment and results. A. N, S. A and H. A have contributed to the partial write-up, visualized the results. All authors reviewed and approved the final version of the manuscript.

## Data Availability

Figshare: Blockchain based secure voting system, https://doi.org/10.6084/m9.figshare.28113140.v1. ^79^ This project contains the following underlying data:
• Polling folder • Readme • Data file – Citizen data_with_national_id.csv The data supporting the findings of this study are available on under the license GPL 3.0+. All other relevant data are included in the manuscript.

## Footnotes

^1^

https://url.us.m.mimecastprotect.com/s/LI24CkRwomf5wBgE7T2fyTGMWiA?domain=figshare.com

^2^

https://docs.nethereum.com/en/latest/

^3^

https://chainspect.app/dashboard
