# Supplementary material for: Blockchain-enhanced electoral integrity: a robust model for secure digital voting systems in Oman
Source: F1000Res. 2025 Sep 12;14:223. Originally published 2025 Feb 19. [Version 3] doi: 10.12688/f1000research.160087.3 (PMC12397734; doi:10.12688/f1000research.160087.3)
Supplement: Peer review history

# Reviewer report: Reviewer response for version 2

**DOI:** 10.5256/f1000research.180515.r400249

**Published:** 2025-08-30

**Authors:** Abdullah Ayub Khan

**Affiliations:** 1 Bahria University Karachi Campus, Karachi, Sindh, Pakistan

The authors present a technical research paper with relevant topic, proper research methodology and potentially good contribution to the field of studies.

 The authors are encouraged to resubmit the paper with more clarity on presented performance assessment metrics with the selected relevant Case studies

 and possible application scenario with assessment metrics. The paper should be written in proper format, figures should fit within the text, use of font

 should be uniform in all paper, as well as references should be updated with most recent results.

 Suggestion and Recommendation:

 1. Authors may elaborate more on the novelty/contribution of their work and how it

 2. Authors need to be specific about their problem statement and the scope of their research.

 3. Abstract: elaborate more on the problem statement, findings, and contributions.

 4. Introduction is not clear. Authors may contribute more towards this.

 Contributes to the literature in the second last paragraph of the introduction clearly.

 5. Thorough proofreading is recommended.

 6. Anfew of the figures are taken from the sources and are not cited properly (such as AI Generated), either they may be cited properly with permissions or may be removed/redrawn.

 7. The conclusion is not clear and needs revision and clarity and alignment with the abstract and title."

 References:

 1. Your references are not listed in good style, as cåitation style is different from one paper to other.

 2. some of your references are not complete please check.

 3. Some citations (references) created in wrong manner (Please follow journal's criteria).

 Authors are encouraged to base on recent references about the current development in blockchain technology. Moreover, technology collaborates with

 other technologies to create new paradigms, such as artificial intelligence, such machine learning, deep learning, with federated learning.

Is the work clearly and accurately presented and does it cite the current literature?

Yes

If applicable, is the statistical analysis and its interpretation appropriate?

Yes

Are all the source data underlying the results available to ensure full reproducibility?

Yes

Is the study design appropriate and is the work technically sound?

Yes

Are the conclusions drawn adequately supported by the results?

Yes

Are sufficient details of methods and analysis provided to allow replication by others?

Yes

Reviewer Expertise:

Blockchain

I confirm that I have read this submission and believe that I have an appropriate level of expertise to confirm that it is of an acceptable scientific standard.

# Reviewer report: Reviewer response for version 2

**DOI:** 10.5256/f1000research.180515.r400248

**Published:** 2025-07-25

**Authors:** Farhan Rafat Khan

**Affiliations:** 1 Cyber Security, Air University, Islamabad, FEDERAL CAPITAL, Pakistan

**Peer Review Comments**

**1. Novelty and Contribution to Knowledge**

 The paper presents a blockchain-based model for enhancing electoral integrity in Oman’s digital voting system. While blockchain for e-voting is not a novel concept, the application to Oman’s electoral context provides regional relevance. The authors contribute by:

- Proposing a **simplified blockchain model** tailored for Oman’s voting system.
- Integrating **biometric authentication** with blockchain to strengthen voter verification.
- Evaluating **asynchronous vs. synchronous transactions** for efficiency.

However, the novelty is somewhat limited, as similar frameworks have been proposed for other countries (e.g., Estonia, South Korea). The paper would benefit from a clearer differentiation from prior work, particularly in addressing **Oman-specific challenges** (e.g., public trust, regulatory readiness).

**2. Technical Contents**

 The technical methodology is well-structured, with:

- A **quantitative experimental approach** using synthetic data.
- **Smart contract implementation** (Ethereum-based) for voter authentication and vote recording.
- **Performance evaluation** on transaction times (registration, voting).

**Concerns:**

- **Scalability:** The paper acknowledges Ethereum’s limitations (~15-30 TPS) but does not sufficiently test alternative Layer-2 solutions (e.g., Rollups, Sharding) for large-scale elections.
- **Security Analysis:** While Sybil and 51% attacks are mentioned, deeper threat modeling (e.g., collusion resistance, side-channel attacks) is needed.
- **Real-world Feasibility:** The synthetic data simulation lacks real-world validation (e.g., stress-testing under adversarial conditions).

**3. Clarity of Work/Presentation**

 The paper is **well-organized**, with clear sections and logical flow. However:

- **Figures (e.g., 6-9):** The transaction time plots are useful but could be better annotated (e.g., error margins, statistical significance).
- **Algorithm Descriptions:** The pseudocode (Algorithms 1-5) is clear but lacks complexity analysis (e.g., gas costs for smart contracts).
- **Jargon:** Some blockchain terms (e.g., "trust-less distributed voting") could be simplified for broader readability.

**4. Referencing**

 The references are **relevant and up-to-date**, covering blockchain, e-voting, and Omani electoral processes. However:

- **Missing Key Works:** No discussion of **Zero-Knowledge Proofs** (e.g., ZK-SNARKs) for privacy-preserving voting, despite their growing relevance.
- **Over-reliance on Theoretical Papers:** More empirical studies (e.g., Estonia’s i-Voting) would strengthen the practical insights.

**5. Comments to Authors**

**Strengths:**

- A **practical blockchain model** with biometric integration.
- **Performance metrics** (async vs. sync transactions) provide actionable insights.

**Areas for Improvement:**

1. **Expand Security Analysis:** Include formal threat modeling (e.g., MITRE ATT&CK for Blockchain).
2. **Test Scalability Solutions:** Evaluate Polygon or Optimistic Rollups for higher TPS.
3. **Address Privacy:** Discuss how voter anonymity is preserved despite blockchain’s transparency.
4. **Compare with Existing Systems:** How does this model improve upon Oman’s current "Sawtak" system?

**6. Comments to Editor**

**Accept with Major Revisions.** The paper has merit but requires:

- **Deeper technical validation** (e.g., live network testing, adversarial simulations).
- **Stronger differentiation** from prior blockchain voting systems.
- **Policy Implications:** More concrete steps for Omani adoption (e.g., pilot deployment challenges).

If revised thoroughly, this could be a valuable contribution to **applied blockchain research in governance**.

**7. Final Decision**

**Conditional Acceptance** – pending revisions addressing scalability, security, and real-world applicability.

Is the work clearly and accurately presented and does it cite the current literature?

Partly

If applicable, is the statistical analysis and its interpretation appropriate?

Partly

Are all the source data underlying the results available to ensure full reproducibility?

Yes

Is the study design appropriate and is the work technically sound?

Partly

Are the conclusions drawn adequately supported by the results?

Partly

Are sufficient details of methods and analysis provided to allow replication by others?

Partly

Reviewer Expertise:

cryptography, steganography, cybersecurity, information security, i-voting, information theory

I confirm that I have read this submission and believe that I have an appropriate level of expertise to state that I do not consider it to be of an acceptable scientific standard, for reasons outlined above.

# Reviewer report: Reviewer response for version 2

**DOI:** 10.5256/f1000research.180515.r382046

**Published:** 2025-06-20

**Authors:** Blerim Rexha

**Affiliations:** 1 Department of Computer Engineering, University of Prishtina, Prishtina, Kosovo, Albania

**Final Assessment: Accept with minor recommendation**

 To make the article scientifically robust and publication-ready, the authors could address the following points:

 - Expand literature review to include recent (2024-2025) studies on Ethereum scalability solutions and smart contract security

 - Perform explicit statistical analyses on transaction data (confidence intervals, standard deviations, significance testing) and clearly report these statistics to provide more rigorous support for the claims.

 - Clearly differentiate between experimentally supported conclusions and speculative statements on infrastructure readiness, societal acceptance, and public trust.

Is the work clearly and accurately presented and does it cite the current literature?

Yes

If applicable, is the statistical analysis and its interpretation appropriate?

Partly

Are all the source data underlying the results available to ensure full reproducibility?

Yes

Is the study design appropriate and is the work technically sound?

Yes

Are the conclusions drawn adequately supported by the results?

Yes

Are sufficient details of methods and analysis provided to allow replication by others?

Yes

Reviewer Expertise:

Cyber security, electronic voting, Blockchain, privacy

I confirm that I have read this submission and believe that I have an appropriate level of expertise to confirm that it is of an acceptable scientific standard.

# Response

**Published:** 2025-09-01

**Authors:** Abdul Shaikh

**Affiliations:** Information Systems Department, Sultan Qaboos University, Muscat, Muscat Governorate, Oman

**Paper Title: “Blockchain-enhanced electoral integrity: a robust model for secure digital voting systems in Oman**

**Responses on Reviewer’s Comments by Authors**

 We would like to thank the expert reviewer for the constructive feedback. We have addressed all the comments and feedback as below.

 We believe now our revised manuscript has significantly improved

**Reviewer 1 Comment #1**

 To make the article scientifically robust and publication-ready, the authors could address the following points:

 Expand literature review to include recent (2024-2025) studies on Ethereum scalability solutions and smart contract security

 Author Response:

 Thank you for this valuable and constructive feedback.

 To strengthen the scientific robustness of the article, we have expanded the literature review by adding eight recent references (2024–2025) relevant to the latest developments in Ethereum scalability and smart contract securityalong with adding of two dedicated subsections under Section 3.0 ‘Related Work’:

 These additions provide a more up-to-date and comprehensive foundation for our study and align the manuscript with the latest state-of-the-art research.

 Reference: Section 3.1 and 3.2 Page # 13-14

**Reviewer 1 Comment #2**

 - Perform explicit statistical analyses on transaction data (confidence intervals, standard deviations, significance testing) and clearly report these statistics to provide more rigorous support for the claims.

 Author Response:

 Due to data availability constraints, the nature of the dataset, limitations (such as cost for using real-world blockchain network for real the transactions (in the data collection process, or the scope of the current study, which is primarily about the blockchain model for online voting, we are unable to perform such analyses at this time. However, we highly appreciate the comments.

**Reviewer 1 Comment #3**

 Clearly differentiate between experimentally supported conclusions and speculative statements on infrastructure readiness, societal acceptance, and public trust.

 Author Response:

 Thank you for this helpful observation. To address this observation, we have revised the manuscript by adding clarifying content in Section 6.6 (Performance Evaluation). In this section, we now clearly distinguish between experimentally validated findings (e.g., high throughput of simulated voter data, secure and immutable recording of votes, prevention of duplicate voting through biometric verification) and speculative statements (e.g., potential for increased public trust, higher voter turnout, and infrastructure readiness). We explicitly state that these societal and infrastructural aspects remain hypotheses requiring real-world testing and large-scale validation, while the technical performance outcomes are firmly supported by our simulation results. This revision ensures a transparent separation between evidence-based conclusions and forward-looking projections.

 Reference: Section 6 Page # 25.

  

- **Is the work clearly and accurately presented and does it cite the current literature?**

Yes

 Author Response:

 Thank you

- **Is the study design appropriate and is the work technically sound?**

Yes

 Author Response:

 Thank you

- **Are sufficient details of methods and analysis provided to allow replication by others?**

Yes

 Author Response:

 Thank you

- **If applicable, is the statistical analysis and its interpretation appropriate?**

Partly

 Author Response:

 Addressed

- **Are all the source data underlying the results available to ensure full reproducibility?**

Yes

 Author Response:

 Thank you

- **Are the conclusions drawn adequately supported by the results?**

Yes

 Author Response:

 Thank you

# Reviewer report: Reviewer response for version 1

**DOI:** 10.5256/f1000research.175905.r369072

**Published:** 2025-03-13

**Authors:** Emilien Paulis

**Affiliations:** 1 University of Luxembourg, Esch-sur-Alzette, Luxembourg

This paper presents a blockchain-based model for enhancing electoral integrity in Oman’s digital voting system. It provides a clear rationale for using blockchain technology, details the implementation methodology, and presents experimental results evaluating the model’s efficiency and security. The topic is highly relevant, given the growing global interest in secure and transparent e-voting solutions. The study contributes to the field by proposing and testing a blockchain-based electoral model, but certain areas require further elaboration and refinement, especially when reading the article from the perspective of a political scientist.

**Conceptual and theoretical framework**

 The paper provides a general discussion of blockchain technology but, to me, it lacks a strong theoretical foundation that connects blockchain with electoral integrity from a governance and democracy perspective. It would benefit from a more explicit discussion of trust in institutions, voter perceptions, and the role of digital transparency in democratic legitimacy.

**Methodology and experimental setting**

- While the experimental setup is described in detail, it is unclear how representative the synthetic data is compared to actual voter data in Oman.
- The scalability of the proposed system is not fully addressed: what happens if the voter base significantly increases? Will Ethereum nodes efficiently handle large-scale elections?
- The performance evaluation primarily focuses on processing time but lacks a deeper analysis of security vulnerabilities, potential attack vectors, or risks of blockchain manipulation.
- The choice of Ethereum should be justified further: are there alternative blockchain platforms that might offer better scalability, efficiency, or security
- How is Oman a particular case (or not) regarding the use of a digital voting system? I'd like the authors to be more reflective about the limit of the case and the external validity of their experimentation.

**Conclusion/implications**

- The conclusion highlights the importance of public trust but does not provide empirical evidence or survey data on how Omani citizens perceive blockchain-based voting. I would at least open this perspective as future research area.
- The claim that Oman could lead global innovation in electoral blockchain technology is ambitious but not fully substantiated—how does this model compare with existing blockchain-based voting systems in other countries?

**Language/writing**

- Some sections, particularly the introduction and related work, contain grammatical errors and awkward phrasing, which could be improved for better readability.
- The citations could sometimes be better integrated. Some seem to be listed without critical engagement or discussion in the text.

Is the work clearly and accurately presented and does it cite the current literature?

Partly

If applicable, is the statistical analysis and its interpretation appropriate?

Not applicable

Are all the source data underlying the results available to ensure full reproducibility?

Yes

Is the study design appropriate and is the work technically sound?

Yes

Are the conclusions drawn adequately supported by the results?

Partly

Are sufficient details of methods and analysis provided to allow replication by others?

Yes

Reviewer Expertise:

Political science

I confirm that I have read this submission and believe that I have an appropriate level of expertise to confirm that it is of an acceptable scientific standard, however I have significant reservations, as outlined above.

# Response

**Published:** 2025-04-14

**Authors:** Abdul Shaikh

**Affiliations:** Information Systems Department, Sultan Qaboos University, Muscat, Muscat Governorate, Oman

**Paper Title: “Blockchain-enhanced electoral integrity: a robust model for secure digital voting systems in Oman**

**Responses on Reviewer’s Comments by Authors**

 We would like to thank the expert reviewer for the constructive feedback. We have addressed all the comments and feedback as below.

 We believe now our revised manuscript has significantly improved

**Reviewer’s Comment**

  

- This paper presents a blockchain-based model for enhancing electoral integrity in Oman’s digital voting system. It provides a clear rationale for using blockchain technology, details the implementation methodology, and presents experimental results evaluating the model’s efficiency and security. The topic is highly relevant, given the growing global interest in secure and transparent e-voting solutions. The study contributes to the field by proposing and testing a blockchain-based electoral model, but certain areas require further elaboration and refinement, especially when reading the article from the perspective of a political scientist.

**Author Response**

 Thank you for the supportive comments.

**Reviewer’s Comment**

**Conceptual and theoretical framework**

  

- The paper provides a general discussion of blockchain technology but, to me, it lacks a strong theoretical foundation that connects blockchain with electoral integrity from a governance and democracy perspective. It would benefit from a more explicit discussion of trust in institutions, voter perceptions, and the role of digital transparency in democratic legitimacy.

**Author Response**

 Reference:

 \subsection{Blockchain with Electoral Integrity from a Governance and Democracy Perspective}

 Blockchain technology has captured their attention as scholars and policymakers seek to bolster integrity in elections. Dominating the conversations was the impact of digital technology on institutional trust and its relation to electoral systems and the confidence voters placed in those systems \cite{b55}. The available literature reveals that implementing blockchain in voting systems has advantages and challenges.

 Defenders of blockchain note that its features, particularly decentralization and the inability to alter information, can remarkably enhance the transparency of elections. Ohize et al. \cite{b52} stress that mobile voting systems based on blockchain technology have the potential to eliminate voter fraud and increase turnout by providing a secure and verifiable system for voting. They highlight that blockchain-based voting systems can be audited by anybody, enhancing credibility and faith in the system and electoral framework. Regardless of these advantages, Assouline et al.\cite{b56} argue that some privacy issues, cybersecurity, digital skills, and the exclusion of certain demographics add to the criticism of using blockchain technology in elections. There needs to be sufficient public awareness and development of the relevant technology to enable everyone’s participation.

 Similarly, Farooq et al.\cite{b53} explain how blockchain can help protect voter registration information by making alterations transparent and traceable. Jafar et al. \cite{b58} discussed that electronic voting machines with blockchain technology could issue a printed paper receipt to the voters, which would aid in audits and boost trust in the vote-counting process. Moreover, Olaniyi et al. \cite{b57} underscore the risks of applying new technologies to the election process. They contend that elections are critical undertakings wherein things cannot go wrong since poorly applied blockchain systems would lose public trust. Implementing any technological application should be preceded by robust public awareness campaigns managed by reputable, non-partisan election officials.

 The use of blockchain in the 2023 elections in Guatemala is an example that illustrates the expected results of the implementation. As stated in a Democracy and Society \cite{b54} report, the technology was applied to restore trust in the electoral system after allegations of fraud in preceding elections. Storing each transaction in a ledger using blockchain technology boosted the electoral system by easily detecting attempted manipulations.

 One of the distinguishing features of democracy is transparency and digital democracy. As noted by many researchers, blockchain technology requires a guarded approach regarding security and public trust \cite{b59}. These two elements demonstrate the utmost necessity in maintaining the public's trust in the system. Transparency of information coupled with accessibility indicates an open, robust form of government, which can be achieved through Internet technology. With innovative ideas purchasable through the internet, civic engagement engages us to welcome and engage in democracy through active voting. The exercise of voting becomes a civic duty for citizens of democratic countries as it incredibly directly sources their choices and decisions.

 In this regard, elections are a striking example of implementing civic engagement. However, they tend to ignore that civic engagement can be defined in a multi-dimensional framework that features systems thinking that captures the complexity of social systems, making a participatory approach more appealing. Regardless of who utilizes the notion of digital democracy alongside open government, the digitally achieved or rather opened democracy acts as a form of power that the government can exercise and aid in refining the understanding of democracy through freedom.

 \subsubsection{Theoretical Foundations and Potential Benefits}

 A blockchain functions as a transparent public ledger incapable of modification, which opens the possibility for a new method of ensuring fair elections. Employing specific coding techniques, voting systems based on blockchain technology can offer End-to-End Verifiable (E2E) elections \cite{b60}. E2E implies that votes are cast on time, midstream, stored, and counted while stored. This capability can resolve problems associated with election fraud, risk of hacking, and personnel error. However, adding blockchain does not automatically provide more trust in elections \cite{b61}. Rather, the trust in blockchain voting is founded on the perception of voters, political leaders, and election officers. Without trust and knowledge of such systems, citizens cannot endorse democracy.

 \subsubsection{Trust in Institutions and Electoral Legitimacy}

 Trust in institutions is one of the central issues in political science. To what degree do people consider that political institutions are executing their roles properly? Standard voting systems dependent on institutions like election commissions, political parties, and independent monitors for free and fair elections rely on multiple functions working synergistically to acquire an equilibrium bloc \cite{b62}. It’s more a question of voters’ trust in the technology than in existing institutions and whether blockchain technology, which offers a mechanism without a need for a central authority, will make freer elections possible.

 Most studies indicate a general decline in confidence in voting systems across the globe. People are apprehensive regarding cheating, vote tampering, and cybersecurity threats \cite{b63,b64,b65}. Blockchain is suggested as an answer to this problem because there is no need to trust a central authority. All the transactions (votes) can be independently verified. Political analysts say elections are primarily social and not purely technical \cite{b66}. If people remain doubtful about blockchain security, ease of use, and the possibility of government tampering, increased reliability of elections will not be guaranteed.

 Research indicates mixed results on users' trust in voting via the Internet. Some suggest that younger, more adept technology users will trust elections run on blockchains while older users grapple with the system’s clarity and transparency. Moreover, in cases where there is extreme political division within parties, new election platforms are likely to be more divisive than unifying.

 \subsubsection{Voter Perceptions and Digital Transparency}

 Public trust in the fairness and accessibility of the electoral processes is fundamental to their credibility. A blockchain framework could worsen fears and misunderstandings without clarity, education, and transparency.

 \begin{itemize}

 \item      \textbf {Perceived Opacity vs. Actual Transparency}

 Although blockchain can improve transparency by allowing voting processes to be publicly verified, most voters lack the technical skills necessary to audit blockchain systems \cite{b67,b68}. Without straightforward verification mechanisms, blockchain implementation could inadvertently generate a black-box scenario where the community must depend on trusted specialists to navigate the opaque system instead of analyzing the results independently.

 \item      \textbf {Digital Literacy and Accessibility}

 The positive impact of blockchain technology on electoral integrity relies on the assumption that all voters have the requisite digital literacy skills to engage with a blockchain voting system. Nonetheless, some studies indicate that the digital divides informed by education, age, and socioeconomic status can extend existing disenfranchisement \cite{b69}. That said, implementing blockchain voting needs to be preceded by efforts to educate voters about the technology so that they have adequate understanding and trust in the system.

 \item      \textbf {Role of Social Media and Disinformation}

 The capability of blockchain technology to secure electoral data does not address the challenges of misinformation. Today's elections are as much about the social media narratives, spin, and misinformation surrounding them as they are about casting votes \cite{b70}. Even if there were a voting system based on blockchain that was utterly impenetrable, trust in the system would still be undermined in highly polarised elections due to rampant inaccurate claims regarding the results.

 \end{itemize}

 \subsubsection{The Role of Digital Transparency in Democratic Legitimacy}

 Democratic legitimacy defines whether a government has the right to rule and if it acts with the consent of the citizens. Improving digital transparency with blockchain technology may help in improving legitimacy by:

 \begin{itemize}

 \item Offering tangible proof that hacking did not occur with the voting.

 \item Minimizing dependence on authoritative sides reduces the chance of bias from the institutions.

 \item Increasing the possibility of international observation of elections, allowing outsiders to witness the processes live.

 \end{itemize}

 Electins conducted using blockchain technology may expose previously undetected discrepancies in the voting process. These discrepancies, however, could be misinterpreted as fraudulent activity, thus eroding trust in the electoral system. While blockchain may reinforce election integrity, its effectiveness will hinge on public perception, institutional support, and integration into current electoral systems \cite{b71}. Should insufficient public confidence and trust exist, an inclusive strategy could lead to opposition to the elections. Lastly, to bolster the democratic image of a nation, issues surrounding democratic election integrity need comprehensive public trust initiatives and civic education campaigns that examine governance issues, establish trust in the system, and systematically approach the integration of blockchain technology.

*Reviewer’s Comment

**Methodology and experimental setting**

- While the experimental setup is described in detail, it is unclear how representative the synthetic data is compared to actual voter data in Oman.

Authors Response

 Reference \section{Methods}

 The research study generated synthetic data for the experimental purpose using Python and code is shared with additional material. Authors ensured its validity and accuracy, as it undergoes rigorous data integrity checks to ensure consistency and reliability. These checks include range validation, ensuring all values fall within acceptable limits, such as reasonable demographic details; format validation, confirming that data structures like national IDs adhere to predefined formats; and presence checks, ensuring all necessary fields are present without omissions. By leveraging these checks, the synthetic data mirrors key attributes of real-world population demographics while maintaining privacy and security. Such validation ensures the data's suitability for modeling the blockchain-based voting system, allowing the experiment to test functionalities and performance metrics effectively while safeguarding against data errors and inconsistencies. This robust validation approach makes the synthetic data both dependable and fit for the study's purposes.

*Reviewer’s Comment

- The scalability of the proposed system is not fully addressed: what happens if the voter base significantly increases? Will Ethereum nodes efficiently handle large-scale elections?

**Authors Response**

 We have updated the following lines in the conclusion section to address the above concern

 Reference \section{Conclusion}

 While the proposed model offers numerous benefits, it also has limitations that need to be considered. For instance, the issue of scalability needs to be addressed as the number of voters increases substantially. Theoretically, the Ethereum Network supports 119.1 transactions per second (tx/s) (footnote 1), [1]which is relatively lower than other blockchain networks, such as Solana (65000 tx/s), Tron (2516 tx/s), Stellar (1137 tx/s), BNB Chain (2222 tx/s), and Polygon (714.1 tx/s). There are various scaling solutions, including sharding, off-chain computation, rollups (such as Optimistic or ZK-Rollups), and hardware acceleration, among others. Future work includes testing scalability and performance across various blockchain networks.

*Reviewer’s Comment

- The performance evaluation primarily focuses on processing time but lacks a deeper analysis of security vulnerabilities, potential attack vectors, or risks of blockchain manipulation.

**Authors Response**

 We have added the following content at the end of the "Result and Discussion" section to address the above concern

 The experiment is primarily conducted to focus on the viability and processing time of election-related transactions. There are security-related issues, such as security vulnerabilities, potential attack vectors, or the risk of blockchain manipulation. While blockchain is widely considered as a secure and tamper-resistant technology, it is susceptible to several potential vulnerabilities and attack vectors such as sybil attacks, 51% attacks, Smart Contract Vulnerabilities, data integrity and oracle manipulation, Denial of Service (DoS) attacks, Key Management and Insider Threats, Blockchain forking and inconsistency (Citation-1), among others ( https://owasp.org/www-project-smart-contract-top-10/). The most pertinent threats are discussed below:

- Sybil Attacks: In a Sybil attack, an adversary creates multiple fake identities to gain disproportionate influence over the network. This can compromise consensus decisions, particularly in permissionless blockchains. It can be mitigated by enforcing large stakes for validators in a network like Ethereum (Citation-2)
- 51% Attacks: A 51% attack occurs when a single entity or coalition controls most of the network’s computational or stake power, allowing them to manipulate the ledger (e.g., double-spending). While this risk is more common in public, proof-of-work systems, we can minimize it by using a practical Byzantine Fault Tolerant (PBFT) consensus algorithm among pre-approved validators, making it resilient against such attacks (Citation-1).
- Smart Contract Vulnerabilities: If smart contracts are used in the framework, they can introduce exploitable bugs (e.g., reentrancy, overflow/underflow errors, or improper access control). We can address this by enforcing rigorous contract auditing and adopting standardized development practices, such as those recommended by OpenZeppelin and formal verification tools when feasible (Footnote for this web link:https://ethereum.stackexchange.com/questions/83542/verifying-erc20-contract-that-uses-openzeppelins-library-contracts ).
- Denial of Service (DoS) Attacks: DoS attacks can target validator nodes or clog the network with excessive transactions. We can implement rate-limiting, transaction prioritization, and resource-monitoring techniques to address this, especially at the application layer (Citation-3).

The choice of Ethereum should be justified further: are there alternative blockchain platforms that might offer better scalability, efficiency, or security

**Authors Response**

 Reference \section{Methods and Results}

 Ethereum offers a highly secure and transparent foundation for electoral processes, ensuring trust in digital voting systems. Its smart contract automation eliminates the need for intermediaries, reducing human errors and minimizing the risk of election fraud \cite{b72}. By leveraging a decentralized network, Ethereum ensures that no single entity controls the voting process, making tampering or manipulation virtually impossible. Additionally, the immutability of its blockchain enhances public confidence by recording every vote on a transparent ledger that cannot be altered.  In terms of scalability, Ethereum Network supports 119.1 transactions per second (tx/s) (footnote 1), which is relatively lower than other blockchain networks, However, there are various scaling solutions, including sharding, off-chain computation, rollups (such as Optimistic or ZK-Rollups), and hardware acceleration, among others. Future work can be include testing scalability and performance across various blockchain networks.

- How is Oman a particular case (or not) regarding the use of a digital voting system? I'd like the authors to be more reflective about the limit of the case and the external validity of their experimentation.

*Reviewer’s Comment

**Conclusion/implications**

- The conclusion highlights the importance of public trust but does not provide empirical evidence or survey data on how Omani citizens perceive blockchain-based voting. I would at least open this perspective as future research area.
- The claim that Oman could lead global innovation in electoral blockchain technology is ambitious but not fully substantiated—how does this model compare with existing blockchain-based voting systems in other countries?

**Author’s response**

 Thank you for your valuable comments

 The conclusion has been revised as below to address the above concern

 It has been concluded that blockchain technology has enhanced the integrity and security of digital voting systems in Oman. Blockchain can significantly bolster voter trust and participation in the electoral process by addressing critical issues such as data integrity, fraud prevention, and transparency. While the advantages of such technology are widely discussed, there is scant evidence detailing public perception, especially about Omani citizens. It is important to know how Omanis view a blockchain-based voting system because public trust is critical to the success of any election reform. Studies conducted in Oman are lacking, and future work should focus on designing effective questionnaires and trust measurement scales that accurately determine voters' trust in the technology. Estonia, where its e-governance systems, including its i-Voting system, were bolstered into blockchain technology. The adoption of blockchain has improved security and transparency, increasing public trust in digital voting. The Estonia example demonstrates the necessity of solid digital frameworks alongside a digitally connected and trusting populace. The blockchain-based voting framework ensures a tamper-proof and transparent voting system, maintains voter privacy, and facilitates real-time auditing. Successful implementation, however, requires overcoming several challenges, including technological readiness, scalability, and societal resistance to change. The study highlights the necessity for a robust regulatory framework and comprehensive public awareness initiatives to foster acceptance and understanding of blockchain technology in the electoral context. Policymakers are urged to initiate pilot projects to evaluate the practical implications of Blockchain integration, ensuring a gradual and informed transition. Ultimately, adopting blockchain in Oman’s electoral system could serve as a model for other nations seeking to enhance the security and credibility of their voting processes. Oman has the opportunity to set a precedent in electoral innovation, ensuring that its democratic processes are robust, transparent, and trustworthy. While the proposed model offers numerous benefits, there are risks and limitations to consider, including the need for advanced infrastructure and technical expertise, potential scalability issues as voter numbers grow, and resistance from the public and stakeholders unfamiliar with or concerned about the reliability of Blockchain technology. Oman must analyze and draw lessons from other global practices to advance to a leading position in blockchain voting innovation. This goes beyond mobilizing the technology and logistics to examine the socio-political landscape of public trust. By conducting basic perception research on citizens, Oman can customize models compared to existing ones to fit its culture and politics. Such an approach would ensure that all technological changes important in elections would work and be endorsed by the people.

**Language/writing**

- Some sections, particularly the introduction and related work, contain grammatical errors and awkward phrasing, which could be improved for better readability.
- The citations could sometimes be better integrated. Some seem to be listed without critical engagement or discussion in the text.

**Author’s response**

 Thank you for your valuable comments

 We have thoroughly revised the introduction and related work sections to correct grammatical errors and improve the overall clarity and flow of the text. Also, we have carefully reviewed and revised the the references and citations throughout the manuscript. Where appropriate, we have added further discussion and critical engagement to ensure that cited works are meaningfully connected to our contributions.

- **Is the work clearly and accurately presented and does it cite the current literature?**

Partly

- **Is the study design appropriate and is the work technically sound?**

Yes

- **Are sufficient details of methods and analysis provided to allow replication by others?**

Yes

- **If applicable, is the statistical analysis and its interpretation appropriate?**

Not applicable

- **Are all the source data underlying the results available to ensure full reproducibility?**

Yes

- **Are the conclusions drawn adequately supported by the results?**

Partly

 Competing Interests

*No competing interests were disclosed.*

 Reviewer Expertise

 Political science

[1] https://chainspect.app/dashboard
